# Stability analysis based parameter tuning of Social Group Optimization

**DOI:** 10.1007/s40747-022-00684-y

**Published:** 2022-02-23

**Authors:** Junali Jasmine Jena, Samarendra Chandan Bindu Dash, Suresh Chandra Satapathy

**Affiliations:** 1 KIIT (Deemed to be University), Bhubaneswar, India; 2 Informatica, Bangalore, India

**Keywords:** Stability analysis, Social Group Optimization, Evolutionary optimization, Parameter tuning

## Abstract

Swarm-based optimization algorithms have been popularly used these days for optimization of various real world problems but sometimes it becomes hard to estimate the associated characteristics due to their stochastic nature. To ensure a steady performance of these techniques, it is essential to have knowledge about the range of variables, in which a particular algorithm always provides stable performance and performing stability analysis of an algorithm can help in providing some knowledge regarding the same. Many researchers have performed the stability analysis of several optimization algorithms and analyzed their behavior. Social Group Optimization (SGO) is a newly developed algorithm which has been proven to yield promising results when applied to many real world problems but in literature no work can be found on stability analysis of SGO. In this paper, Von Neumann stability analysis approach has been used for performing stability analysis of Social Group Optimization (SGO) to analyze the behavior of its algorithmic parameters and estimate the range in which they always give stable convergence. The results obtained have been supported by sufficient experimental analysis and simulated using eight benchmark function suite along with their shifted and rotated variations which prove that the algorithm performs better within the stable range and hence convergence is ensured.

## Introduction

In many real life applications, many complex optimization problems are faced which needs effective solutions. These functions have multiple maxima, minima and saddle points, thus gradient descent/ascent based techniques fail to find the proper solution or most likely lead to a local minima or maxima. For finding the global best value efficient searching of the entire search space becomes necessary. There are several categories of algorithm that fulfill this need. The prominent of them is swarm optimization in which the search space is filled with swarm members with random initial solutions. These members interact with each other (defined by the algorithm), through a series of exploration and exploitation, finally attaining the global best location. Some examples of such algorithms are Particle Swarm Optimization (PSO) [1] which emulates flocking behavior of birds, Artificial Bee Colony (ABC) [2] which emulates behavior of bees, Social Group Optimization (SGO) [3] which emulates human learning pattern of, Grey Wolf Algorithm (GWA) [5] which emulates hunting patterns of Grey Wolves etc. Social Group Optimization (SGO) is a fairly new addition to the shelve. Nonetheless it is very promising addition as it has better convergence rate as compared to many evolutionary algorithms [4]. It has been successfully applied to segmentation of skin melanoma images [6], segmentation of brain MRI images [7], task scheduling in cloud [8], brain tumor evaluation tool [9], automated detection of COVID-19 infection [10], Transformer fault analysis [16], Antenna array synthesis [17], short-term hydrothermal scheduling [18], structural health monitoring in civil engineering [19], solving Travelling Salesman problem [20], solving multi-objective problems [21] and many more.

One essential requirement to be kept in mind while working with these heuristic algorithms is to ensure that the error values remain bounded in a region and don’t explode. To achieve this requirement, stability analysis of an optimization algorithm is essential as it provides the stable range of parameters that prevents the errors from growing beyond boundary making the algorithm converge faster to provide desired solution. In many of the works found in literature, it is observed that the efficacy of an algorithm is claimed by experimentation, but that experimental analysis is subject to that specific problem or situation. Stability analysis is such a method which analyzes the robustness of a system and ’Von Neumann’ method is a suitable method which have been popularly used for this task for swarm optimization methodologies. Various such works can be found in the literature. Gopal et al. [11] have worked on stability analysis of PSO, Bansal et al. [12] in their work performed stability analysis of Differential Evolution, Nair et al. [13] analyzed the stability of Artificial Bee Colony, Farivar et al. [14] have reported on stability analysis of Gravitational Search Optimization, Biswas et al. [15] have analyzed the stability of Bacteria Foraging Optimization etc. To the best of authors’ knowledge the stability analysis of SGO algorithm is not yet attempted. In this paper, authors have addressed the problem of stability of SGO through ‘Von Neumann’ stability criterion and have found stable ranges of parameters for the algorithm.

The rest of the paper is organized as follows. In the second section introduces the SGO algorithm and effects of its update equations on swarm members, in the third section provides details of the Von Neumann Stability Analysis procedure and explains stability analysis in context of swarm optimization. The mathematical analysis of SGO algorithm is carried out in the fourth section and the final section presents the results of simulation based experiments to demonstrate the simulation based find ups.

## Social Group Optimization

Social Group Optimization (SGO) algorithm is a population based distributed optimization algorithm that simulates human learning pattern to iteratively search and reach the global maximum or minimum. It has two distinct phases i.e. improving phase and acquiring phase that execute sequentially in each iteration to exploit and explore the search region respectively.

### Initialization

The initial position of the each member in the population is generated by

2.1 $$\begin{aligned}&A_{i,j} = A_{j_\mathrm{min}} + \mu \left( A_{j_\mathrm{max}}-A_{j_\mathrm{min}}\right) ,\nonumber \\&i=1,2,\ldots ,N, \text { }j=1,2,\ldots ,D, \end{aligned}$$

Here $A_i$ denotes position of the $i\mathrm{th}$ member in the *D* dimensional space. $A_{i,j}$ denotes the $j\mathrm{th}$ dimension of the position vector. $A_{j_\mathrm{max}}$ $A_{j_\mathrm{min}}$ are the upper and the lower bounds of $j\mathrm{th}$ parameter. *N* is the population size and *D* is the dimensionality of the optimization problem under consideration.

### Improving phase

In this phase each swarm member $X_i$ is updated as per Eq. (2.2),

2.2 $$\begin{aligned} A_{\mathrm{new}_{i,j}} = c*A_{\mathrm{old}_{i,j}} + r*(\mathrm{gbest}_{j}-A_{\mathrm{old}_{i,j}}) \end{aligned}$$

Here *c* is the self introspection factor and *r* is a random number. Then $A_{\mathrm{new}_{i,j}}$ is compared with $A_{i,j}$ and the one who provides better performance is accepted as value of $A_{i,j}$.

This equation has a clumping effect on the swarm members as the values of $A_{i,j}$ will be remapped randomly in the connected areas defined by the 4 line segments given in Eqs. (2.3a) to (2.3d) where $r\in [r_n,r_x]$. Eqs. (2.3a) and (2.3b) represent the update equation in improving phase (Eq. (2.2)) when value of *r* is $r_x$ and $r_n$ respectively. Equations (2.3c) and (2.3d) represent the boundary condition $x_\mathrm{min}$ and $x_\mathrm{max}$ defined by the optimization problem.

2.3a $$\begin{aligned} y&= (c-r_x)*x + r_x*\mathrm{gbest}, \end{aligned}$$

2.3b $$\begin{aligned} y&= (c-r_n)*x + r_n*\mathrm{gbest}, \end{aligned}$$

2.3c $$\begin{aligned} x&= x_\mathrm{min}, \text {and } x = x_\mathrm{max} \end{aligned}$$

2.3d $$\begin{aligned} y&= x_\mathrm{min}, \text { and } y = x_\mathrm{max} \, \, \mathrm{(due\,\, to\,\, boundary\,\, constraint)} \end{aligned}$$

**Fig. 1:**
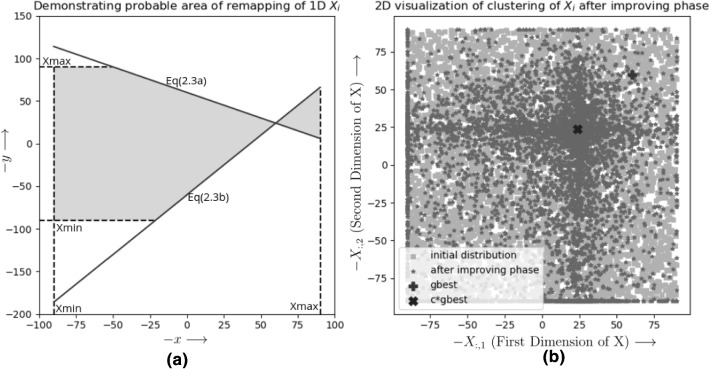
**a** Mapping of Xi in improving phase, **b** clustering in improving phase after first iteration (created with $c,r_n,r_x,x_\mathrm{min},x_\mathrm{max},\mathrm{gbest}$ values as $0.4,-\,1,1,\,-90,90,60$ respectively)

If we observe Eqs. (2.3a) and (2.3b), they intersect at the point $c*\mathrm{gbest}$ as shown in Fig. 1a. Thus, $c*\mathrm{gbest}$ is the cluster centroid. The cluster centroid is formed on the line connecting the origin and gbest. So smaller *c* value always forms the cluster closer to the origin. From Fig. 1a it is clear that for any value of $X_i$ in phe the range, there is always a possibility that it gets mapped to $c*\mathrm{gbest}$ (Can be verified by taking a vertical cross section of the grey area shown in Fig. 1a) and the farther a point is from the centroid on *y*-axis, the range of values of $X_i$ which gets mapped to the value decreases. As the vertical cross-section of the probable area (Grayed portion in Fig. 1a) indicates probability of *y* falling in the region, most points will clump around the cluster centroid. In Fig. 1, the graphical representation of transformation done by Improving phase is shown. The Fig. 1a shows the area defined by the Eqs. (2.3a)–(2.3d) and how the range of $X_i$ is reduced along each dimension(projection on x-axis shows the initial range, and projection on *y*-axis shows the range after improving phase). The Fig. 1b shows a 2D visualization of clustering of $X_i$ after improving phase.

The spread of the cluster roughly can be determined by the angle between the lines reddenoted by $\phi$. It is defined by Eqs. 2.3a and 2.3b. It is controlled by values of *c* and *r* as

$$\begin{aligned}&\mathrm{tan}(\phi ) = \frac{m_1-m_2}{1+m_1m_2}, \text {where } m_1,m_2 \text {are slopes of lines}\\&\text {defined by Eqs. (12.3a) and (12.3b)}~respectively\\&\quad \implies \mathrm{tan}(\phi ) = \frac{(c-r_n)-(c-r_x)}{1+(c-r_n)*(c-r_x)}\\&\quad \implies \mathrm{tan}(\phi ) = \frac{r_x-r_n}{1+c^2-(r_x+r_n)*c+r_nr_x} \end{aligned}$$

Hence the angle can be found as

2.4 $$\begin{aligned} \phi = \mathrm{tan}^{-1}\left( \frac{r_x-r_n}{1+c^2-(r_x+r_n)*c+r_nr_x} \right) \end{aligned}$$

### Acquiring phase

In acquiring phase, each swarm member $A_i$ moves in the search space with respect to gbest and another randomly selected swarm member $A_s$. The update equation is given by
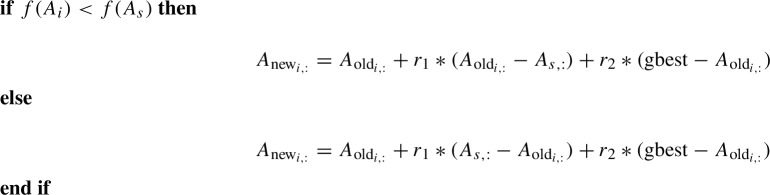


Here $A_s$ is a member of the swarm s.t. $i \ne s$ and $r_1,r_2 \ge 0$. The update equations in acquiring phase can be written as

$$\begin{aligned}&A_{new_{i}} = A_i + (r_2\pm r_1)\left( \frac{r_2*gbest \pm r_1*A_{s}}{r_2 \pm r_1} - A_i\right) \\&\quad \implies A_{new_{i}} = A_i + r_n(A_n - A_i),\\&\text {where } A_n=\frac{r_2*\mathrm{gbest} \pm r_1*A_{s}}{r_2 \pm r_1}\text { and }r_n = r_2 \pm r_1 \end{aligned}$$

**Fig. 2:**
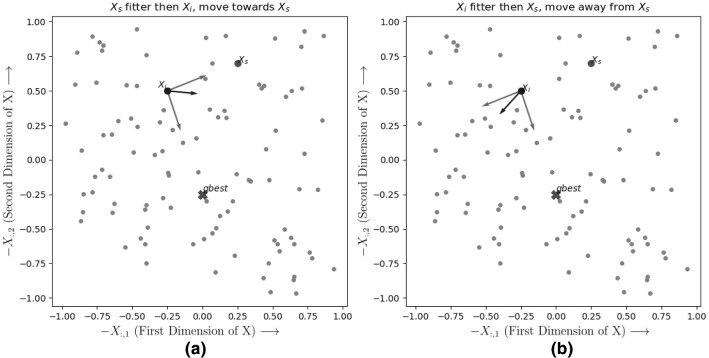
**a** New Position of *Xi* is towards *Xs*. **b** New position of *Xi* is further from *Xs*

So $A_i$ updates it’s position with respect to a vector which is weighted mean of $A_s$ and the *gbest*. Figure 2 shows the direction of movement of $A_i$ in acquiring phase.

So every swarm member updates itself by moving towards or away from another swarm member while moving itself slightly closer to gbest.

## Stability analysis

The stability analysis is an important requirement of numerical estimation schemes involving partial differential equations. In the pre-text context the stability reflects that the total error of a numeric scheme remains bounded. In other words, if some error is introduced it would remain under a bound and would not explode or increase beyond a limit. The Von Neumann stability analysis, which uses Fourier Decomposition, is an important technique, which is chosen to analyse the stability of a scheme.

### von Neumann stability analysis

Consider the partial differential equation represents a system *X*,

3.1 $$\begin{aligned} \frac{\delta X}{\delta \tau } + a \frac{\delta X}{\delta \chi } = 0 \end{aligned}$$

a function of $\chi$ and $\tau$, which evolves through space ($\chi$) and time($\tau$). The numeric estimate of the corresponding partial differential equation is given by red

3.2 $$\begin{aligned}&\frac{X(\tau + \Delta \tau ,\chi )-X(\tau - \Delta \tau ,\chi )}{2*\Delta \tau } \nonumber \\&\quad + a*\frac{X(\tau ,\chi + \Delta \chi )-X(\tau ,\chi - \Delta \chi )}{2*\Delta \chi } = 0 \end{aligned}$$

Equation (3.2) shows numeric estimate of Eq. (3.1) The Fourier series expansion of $X(\tau ,\chi )$ can be written as

3.3 $$\begin{aligned} X(\tau ,\chi ) = \sum _{\begin{array}{c} i\in {1,\ldots ,b_1},\\ t \in {1,\ldots ,b_2} \end{array}} A_me^{-\hbox {J}\beta _m t\Delta \tau }e^{\hbox {J}\alpha _m i \Delta \chi } \end{aligned}$$

where $\chi = i\Delta \chi$, $\tau = t\Delta \tau$. $\Delta \chi$ and $\Delta \tau$ represent the intervals that were used to sample values along the $\chi$ and $\tau$ axis.

Let ${\hat{X}}(t,i)$ represent an estimate of $X(\tau ,\chi )$ with an error $\epsilon _{t,i}$as shown in Eq. (3.4).

3.4 $$\begin{aligned} X(\tau ,\chi ) = {\hat{X}}(t,i) + \epsilon _{t,i} \end{aligned}$$

Here we want to learn whether the error associated with the estimate grows or shrinks with each iteration. So we assume that the error is associated with some components of the Fourier expansion of *X*. We replace *X* in the numeric estimate is replaced by its fourier component to check whether contribution of a single fourier component increases or diminishes over time. If it diminishes then the error associated with the estimate decreases with time too. If the relation between any Fourier coefficient in $t\mathrm{th}$ iteration with the corresponding Fourier coefficient in $(t+1)\mathrm{th}$ iteration can be represented by the Eq. (3.5) and $|g(\alpha _m)| \le 1$ then contribution of each Fourier component decreases with each iteration. So any error associated with it will also diminish over time and we will conclude that the numeric scheme is stable.

3.5 $$\begin{aligned} A_me^{-\hbox {J}\beta _m (t+1)\Delta \tau } = A_me^{-\hbox {J}\beta _m t\Delta \tau }*g(\alpha _m) \end{aligned}$$

### Stability Analysis in context of swarm optimization

In swarm optimization algorithms the update equation is the form

3.6 $$\begin{aligned} A_i^{t+1} = c*A_i^t + f(A_i^t,A_r^t) \end{aligned}$$

Here $A_r$ is another member of the swarm besides $A_i$. $A_i$ interacts with another member of the swarm to update it’s value over each iteration. To determine if it is stable we would want to know if some error gets introduced in an iteration then it diminishes or increases with each subsequent iteration.

For the stability analysis *A* is modelled as a continuous variable across space and time, where each iteration and each member of the swarm is taken as samples at $\tau = t\Delta \tau$ and $\chi = i\Delta \chi$. i.e.

$$\begin{aligned} A_i^t = A(t\Delta \tau ,i \Delta \chi ) = A(\tau ,\chi ) \end{aligned}$$

**Fig. 3:**
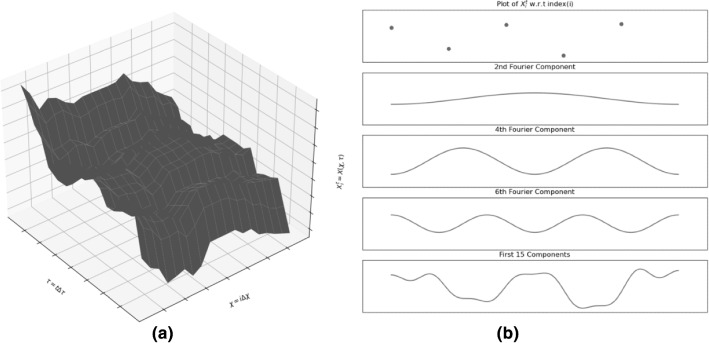
**a** Representation of *A* as continuous variable from it’s grid samples. **b** Fourier decomposition of $A_i$

So all the distinct members of the swarm and each subsequent iteration is modelled as a single continuous function.

This allows us to find a Fourier series corresponding to $A_i^t$ as Eq. (3.7)

3.7 $$\begin{aligned} A_i^t =&A(t\Delta \tau ,i\Delta \chi ) = A(\tau ,\chi )\nonumber \\ {}&\quad = \sum _{\begin{array}{c} i\in {1,\ldots ,b_1}, t \in {1,\ldots ,b_2} \end{array}} A_me^{-\hbox {J}\beta _m t\Delta \tau }e^{\hbox {J}\alpha _m i \Delta \chi } \end{aligned}$$

Now we can do von Neumann stability analysis on the update equation (Eq. (3.6)) by replacing $A_i^t$ with it’s Fourier component $A_me^{-\hbox {J}\beta _m t\Delta \tau }e^{\hbox {J}\alpha _m i \Delta \chi }$. We would get a equation of the form

$$\begin{aligned}&A_me^{-\hbox {J}\beta _m (t+1)\Delta \tau } = A_me^{-\hbox {J}\beta _m t\Delta \tau } * g(\alpha _m) \\&\quad \implies e^{-\hbox {J}\beta _m \Delta \tau } = g(\alpha _m) \end{aligned}$$

If $e^{-\hbox {J}\beta _m \Delta \tau }$ lies inside the unit circle in the Hilbert Space (complex space) then with each update the contribution of a single Fourier component would diminish over time. So the condition of stability is given as

3.8 $$\begin{aligned} |g(\alpha _m)| \le 1 \implies |e^{-\hbox {J}\beta _m \Delta \tau }| \le 1 \end{aligned}$$

### Optimization functions

Table 1 lists eight standard objective functions to be optimized along with their shifted and rotated version. For this the steps mentioned in the CEC 2014 [22] is followed. The functions are mentioned under the column objective function of Table 1 and are expressed as a function of *z*. But for better control and more variety we define *z* as a function of *x*. So we can shift,scale and rotate *x* vectors before passing them into the functions.

The mappings from *x* to *z* are defined under the column **z**. Different mappings from *x* to *z* are defined to generalize the properties as stated-

Range of A: $$x_j \in [-R,R]$$

Global Best value location: $\mathrm{gbest} = o$

and *M* is a $D\text {x}D$ rotation matrix. In this paper we will keep $R=100$ as per CEC 2014 [22] and we will use different values of *M*,*o* and *D* to test the algorithm.

**Table 1:** List of optimization functions

Name	z	Objective function
Sphere	$z = M*(x-o)$	$\mathrm{Min}f_1(x)=\sum _{j=1}^{D}z_j^2$
Rosenbrock	$z = M*\left( \frac{2.048(x-o)}{R}+1 \right)$	$\mathrm{Min}f_2(x)=\sum _{j=1}^{D-1}(100(z_{j+1}-z_{j}^2)^2+(z_{j}-1)^2)$
Ackley	$z = M*\left( \frac{32(x-o)}{R}\right)$	$$\begin{array}{l c l} \mathrm{Min}f_3(x) &{}=&{} -20*exp\left( -0.2*\sqrt{\frac{1}{D}\sum _{j=1}^{D}z_j^2}\right) \\ &{} &{} -exp\left( \frac{1}{D}\sum _{j=1}^{D}\mathrm{cos}(2\pi z_j)\right) +20+ e \\ \end{array}$$
Griewank	$z = M*\left( \frac{600(x-o)}{R}\right)$	$\mathrm{Min}f_4(x)=\sum _{j=1}^{D}\frac{z_j^2}{4000} - \prod _{j=1}^{D}\mathrm{cos}\left( \frac{z_j}{\sqrt{j}}\right) + 1$
Rastrigin	$z = M*\left( \frac{5.12(x-o)}{R}\right)$	$\mathrm{Min}f_5(x)=10D + \sum _{j=1}^{D}(z_j^2-10\mathrm{cos}(2\pi z_j))$
Alpine	$z = M*\left( \frac{10(x-o)}{R}\right)$	$\mathrm{Min}f_6(x)=\sum _{j=1}^{D}(|z_j \mathrm{sin}(z_j) + 0.1*z_j|)$
Sum of powers	$z = M*\left( \frac{(x-o)}{R}\right)$	$\mathrm{Min}f_7(x)=\sum _{j=1}^{D}|z_j^{j+1}|$
Zakharov	$z = M*\left( \frac{10(x-o)}{R}\right)$	$\mathrm{Min}f_8(x)=\sum _{j=1}^{D}z_j^2 + \left( \sum _{j=1}^{D}0.5jz_j \right) ^2 + \left( \sum _{j=1}^{D}0.5jz_j \right) ^4$

## Stability analysis of SGO algorithm

### Approach for a solution

In SGO algorithm we have two phases that operate sequentially in a single epoch which gives the updated value of $A_{\mathrm{new}_i}$ for the next epoch. The equations governing the improving phase is,

4.1 $$\begin{aligned} A_{\mathrm{imp}_{i,j}} = c*A_{i,j} + r*(\mathrm{gbest}_j-A_{i,j}) \end{aligned}$$

and acquiring phase is,

4.2 $$\begin{aligned} A_{\mathrm{acq}_{i,j}} =&A_{\mathrm{imp}_{i,j}} \pm r_1*(A_{s,j}-A_{\mathrm{imp}_{i,j}}) \nonumber \\&+ r_2(\mathrm{gbest}_j-A_{\mathrm{imp}_{i,j}}) \end{aligned}$$

$A_{\mathrm{acq}_i}$ the value of $A_{\mathrm{new}_i}$ for the next iteration. The update equation can be written as

4.3 $$\begin{aligned} \implies A_{\mathrm{new}_{i,j}} =&(c-r)*(1\mp r_1-r_2) A_{i,j} \pm r_1*A_{s,j} \nonumber \\&+ (r_2 + (1\mp r_1-r_2)*r) \mathrm{gbest}_j \end{aligned}$$

Equation (4.3) is a four dimensional equation w.r.t to the parameters $c,r,r_1,r_2$. This equation is very difficult to solve as von Neumann analysis won’t provide enough inequalities to perfectly define ranges of all the parameters. Moreover we will get equations where range of *c*, *r* is dependent on $r_1,r_2$ and vice versa, which is not desirable as both improving and acquiring phases are independent of each other. But we can use a simple trick to simplify these issues. In Eq. (4.1), let’s assume $A_{\mathrm{imp}_{i,j}}=A_{i,j}^{t+1}$ and replace $A_{i,j}^t$ and $A_{i,j}^{t+1}$ with the corresponding Fourier component. We would get an equation of the form,

4.4 $$\begin{aligned} A_me^{-\hbox {J}\beta _m (t+1)\Delta \tau } = A_me^{-\hbox {J}\beta _m t\Delta \tau }*g(\alpha _{m1}) \end{aligned}$$

Doing the same for acquiring phase we would get,

4.5 $$\begin{aligned} A_me^{-\hbox {J}\beta _m (t+1)\Delta \tau } = A_me^{-\hbox {J}\beta _m t\Delta \tau }*g(\alpha _{m2}) \end{aligned}$$

Fourier representation of Eq. (4.3) can be expressed as,

4.6 $$\begin{aligned} A_me^{-\hbox {J}\beta _m (t+1)\Delta \tau } = A_me^{-\hbox {J}\beta _m t\Delta \tau }*g(\alpha _{m1})*g(\alpha _{m2}) \end{aligned}$$

and the corresponding condition for stability is

$$\begin{aligned} |g(\alpha _{m1})*g(\alpha _{m2})| \le 1 \end{aligned}$$

But if

$$\begin{aligned} |g(\alpha _{m1})| \le 1 \end{aligned}$$

and

$$\begin{aligned} |g(\alpha _{m2})| \le 1 \end{aligned}$$

are satisfied, then the condition of stability will be automatically satisfied. Therefore the stability analysis of improving phase and acquiring phase independently can be taken up to obtain the ranges for $c,r,r_1,r_2$.

### Stability analysis

#### Improving phase

The Improving Phase has 2 parameters that govern the equation, *c* and *r*. *c* is the self introspection parameter which is constant, i.e. its value stays the same throughout the algorithm. *r* is a random number. The update equation is given in Eq. (4.7)

4.7 $$\begin{aligned} A_{\mathrm{new}_{i,j}} = c*A_{\mathrm{old}_{i,j}} + r*(\mathrm{gbest}_{j}-A_{\mathrm{old}_{i,j}}) \end{aligned}$$

By replacing $A_{\mathrm{new}_{i,j}}$ and $A_{\mathrm{old}_{i,j}}$ with $A_{i,j}^{t+1}$ and $A_{i,j}^{t}$ respectively, Eq. (4.7) can be rewritten as Eq. (4.8).

4.8 $$\begin{aligned} A_{i,j}^{t+1} = c*A_{i,j}^{t} + r* \left( \mathrm{gbest}_{j}-A_{i,j}^t \right) \end{aligned}$$

In the update equation the dimensions are independent of each other, we can drop j and assume it to be an one dimensional problem without loss of generality. So the Eq. (4.8) reduces to Eq. (4.9).

4.9 $$\begin{aligned} A_{i}^{t+1} = c*A_{i}^{t} + r* \left( \mathrm{gbest}-A_{i}^t \right) \end{aligned}$$

Here the superscript t denotes the value at $t\mathrm{th}$ iteration. For simplifying our calculations we can take *gbest* as a constant. This decision is justified by the fact that after some initial iterations the value of *gbest* updates only occasionally through the algorithm run.

As gbest is a constant we can remove the term from the calculations for stability analysis as there are no errors associated with a constant value. Mathematically, the $m\mathrm{th}$ Fourier component of a constant term is 0 as the Fourier series of the constant is represented by the constant itself.

Replacing $A_i^t$ with it’s $m\mathrm{th}$ Fourier component $A_me^{-\hbox {J}\beta _m t\tau }e^{\hbox {J}\alpha _m i\chi }$ (where $\hbox {J}$ is the imaginary unit) in the Eq. (4.9) an equation similar to the form Eq. (3.5) is obtained.

$$\begin{aligned}&A_me^{-\hbox {J}\beta _m (t+1)\Delta \tau }e^{\hbox {J}\alpha _m i\Delta \chi } = (c-r)*A_me^{-\hbox {J}\beta _m t\Delta \tau }e^{\hbox {J}\alpha _m i\Delta \chi } \\&\quad \implies e^{\hbox {J}\beta _m\Delta \tau } = (c-r) \end{aligned}$$

As per von neumann stability criterion(given in Eq. (3.8)) $|e^{\hbox {J}\beta _m\Delta \tau }| \le 1$. Upon solving the inequality we get,

$$\begin{aligned}&|c-r| \le 1 \\&\quad \implies -1 \le c-r \le 1 \\&\quad \implies c-1 \le r \le c+1 \end{aligned}$$

We obtain the range of *r* as defined in Eq. (4.10). The range of *r* is dependent on *c*.

4.10 $$\begin{aligned} c-1 \le r \le c+1 \end{aligned}$$

**Slope:** For $r_n=c-1$ and $r_x=c+1$ as $r\in [c-1,c+1]$, it can be found that

4.11a $$\begin{aligned}&r_x-r_n = 2 \end{aligned}$$

4.11b $$\begin{aligned}&r_x+r_n = 2c \end{aligned}$$

4.11c $$\begin{aligned}&r_xr_n = c^2 - 1 \end{aligned}$$

Putting these values defined in Eqs. (4.11a)–(4.11c) in Eq. (2.4), we get

4.12 $$\begin{aligned} \phi&= \mathrm{tan}^{-1} \left( \frac{2}{1+c^2-2c^2+c^2-1} \right) \end{aligned}$$

4.13 $$\begin{aligned} \phi&= \frac{\pi }{2} \end{aligned}$$

Therefore, if we use the complete stable range for improving phase, the angle of spread has to be $\pi /2$.

*Experimental results* To verify the findings, the acquiring phase is removed from SGO algorithm and only the improving phase is considered. The stable range of *r* against the three schemes of unstable ranges is compared. They are

1. $$r\in [c+1,c+3]$$ (*r* all positive)
2. $$r\in [-c-3,-c-1]$$ (*r* all negative)
3. $$r\in [-c-2,-c-1]\cup [c+1,c+2]$$

**Fig. 4:**
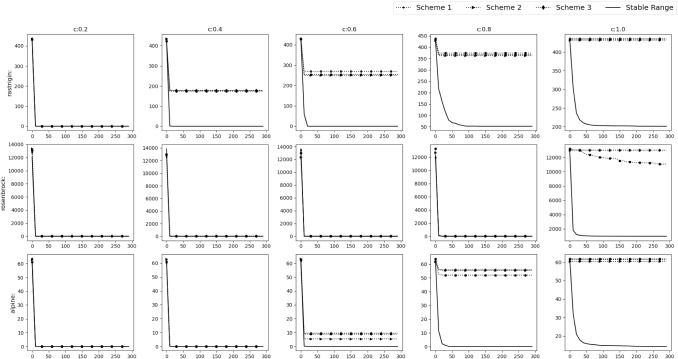
Experimental results of testing of the improving phase with normal functions

**Fig. 5:**
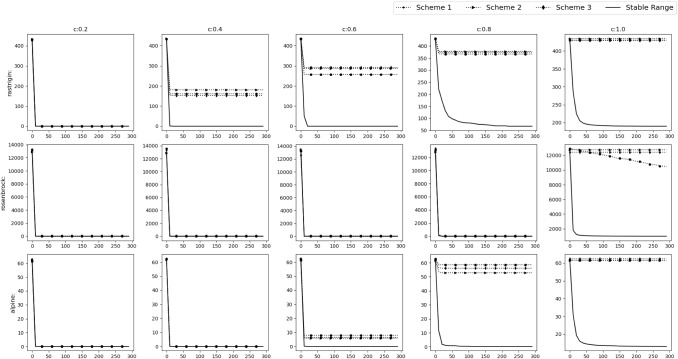
Experimental results of testing of the improving phase with shifted functions

**Fig. 6:**
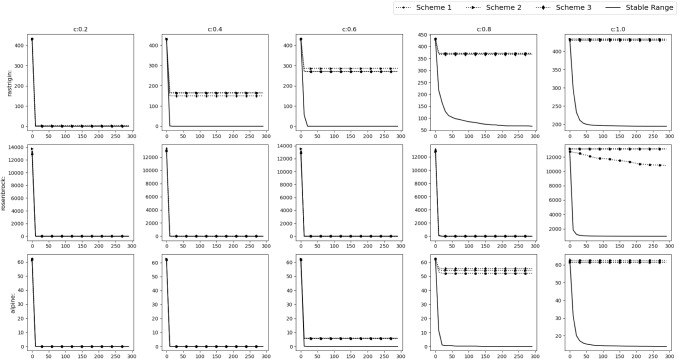
Experimental results of testing of the improving phase with shifted+rotated functions

Rastrigin, Rosenbrock and Alpine function are used for the testing with $D=30$, number of swarm members i.e. $N=50$ with 300 epochs (Or $50*300=15{,}000$ function evaluations). Testing is performed in 3 phases.

- *M*,*o* as identity matrix and null vector respectively. Average taken over 30 iterations.
- *M* as identity matrix and *o* as a random vector whose value will be changed after each 10th iteration. Average taken over 100 iterations.
- *M* as random rotation matrix and *o* as a random vector. Their values will be changed after each 12th iteration. Average and Standard deviation taken over 120 iterations.

Figures 4, 5 and 6 show the results of the 3 phases mentioned above. From the figures it can be observed that, as *c* value increases the stable range performs better than the other 3 unstable schemes.

#### Acquiring phase

The acquiring phase is governed by two parameters i.e. $r_1$ and $r_2$. In acquiring phase, if $A_i$ is fitter than $A_s$, then $A_i$ is updated as

4.14 $$\begin{aligned} A_{\mathrm{new}_{i,:}} =&A_{\mathrm{old}_{i,:}} + r_1*(A_{\mathrm{old}_{i,:}}-A_{s,:}) \nonumber \\&+ r_2*(\mathrm{gbest}-A_{\mathrm{old}_{i,:}}) \end{aligned}$$

else it is updated as

4.15 $$\begin{aligned} A_{\mathrm{new}_{i,:}} =&A_{\mathrm{old}_{i,:}} + r_1*(A_{s,:}-A_{\mathrm{old}_{i,:}}) \nonumber \\&+ r_2*(\mathrm{gbest}-A_{\mathrm{old}_{i,:}}) \end{aligned}$$

Here $A_s$ is a candidate solution such that $i \ne s$ and $r_1,r_2 \ge 0$. We start with Eq. (4.15). The equation can be rewritten as

4.16 $$\begin{aligned} A_{i,:}^{t+1} = A_{i,:}^t + r_1*(A_{s,:}^t-A_{i,:}^t) + r_2*(\mathrm{gbest}-A_{i,:}^t) \nonumber \\ \end{aligned}$$

Here the superscript t denotes the value at $t\mathrm{th}$ iteration. Replacing

$$\begin{aligned} A_s {=} A_{i\pm a}, \text {where } a\in \{\pm 1,\pm 2,\ldots \} \text { and } 1\le i\pm b \le N \end{aligned}$$

as $A_s$ is a member of the swarm and $s\ne i$, we get

4.17 $$\begin{aligned} A_{i,:}^{t+1} = A_{i,:}^t + r_1*(A_{i\pm a,:}^t-A_{i,:}^t) + r2*(\mathrm{gbest}-A_{i,:}^t)\nonumber \\ \end{aligned}$$

In the update equation the dimensions are independent of each other. So it can be assumed as an one dimensional problem without loss of generality. Then the Eq. (4.17) can be rewritten as

4.18 $$\begin{aligned} A_i^{t+1} = A_i^t + r_1*\left( A_{i\pm a}^t-X_i^t \right) + r2*(\mathrm{gbest}-A_i^t)\nonumber \\ \end{aligned}$$

Similar to improving phase, *gbest* can be removed from the stability analysis calculations of acquiring phase, as it being a constant won’t contribute to the errors.

Replacing $A_i^t$ with it’s $m\mathrm{th}$ Fourier component $A_me^{-\hbox {J}\beta _m t\Delta \tau }e^{\hbox {J}\alpha _m i\Delta \chi }$ we get

4.19 $$\begin{aligned}&A_me^{-\hbox {J}\beta _m (t+1)\Delta \tau }e^{\hbox {J}\alpha _m i\Delta \chi } = (1-r_2)*\nonumber \\&\quad A_me^{-\hbox {J}\beta _m t\Delta \tau }e^{\hbox {J}\alpha _m i\Delta \chi } + r_1*(A_me^{-\hbox {J}\beta _m t\Delta \tau }e^{\hbox {J}\alpha _m (i\pm a)\Delta \chi }\nonumber \\&\quad - A_me^{-\hbox {J}\beta _m t\Delta \tau }e^{\hbox {J}\alpha _m i\Delta \chi }) \end{aligned}$$

Upon simplifying we get,

$$\begin{aligned}&A_me^{-\hbox {J}\beta _m (t+1)\Delta \tau }e^{\hbox {J}\alpha _m i\Delta \chi }\\&\quad = (1-r_2)*A_me^{-\hbox {J}\beta _m t\Delta \tau }e^{\hbox {J}\alpha _m i\Delta \chi } + r_1\\&\qquad *(A_me^{-\hbox {J}\beta _m t\Delta \tau }e^{\hbox {J}\alpha _m (i\pm a)\Delta \chi } - A_me^{-\hbox {J}\beta _m t\Delta \tau }e^{\hbox {J}\alpha _m i\Delta \chi }) \\&\qquad \implies A_me^{-\hbox {J}\beta _m (t+1)\Delta \tau }e^{\hbox {J}\alpha _m i\Delta \chi }\\&\quad = (1-r_1-r_2)*A_me^{-\hbox {J}\beta _m t\Delta \tau }e^{\hbox {J}\alpha _m i\Delta \chi } + r_1\\&\qquad *A_me^{-\hbox {J}\beta _m t\Delta \tau }e^{\hbox {J}\alpha _m (i\pm a)\Delta \chi } \\&\qquad \implies e^{-\hbox {J}\beta _m \Delta \tau } = (1-r_1-r_2) + r_1*e^{\hbox {J}\theta },\\&\qquad \text {where } \theta = \alpha _m(\pm a)\Delta \chi \end{aligned}$$

As per Von Neumann stability criterion, $|e^{\hbox {J}\beta _m\Delta \tau }| \le 1$. So,

$$\begin{aligned}&\implies |(1-r_1-r_2) + r_1*e^{\hbox {J}\theta }| \le 1 \\&\quad \implies \sqrt{(1-r_1-r_2+r_1 \mathrm{cos}\theta )^2 + (r_1\mathrm{sin}\theta )} \le 1 \\&\quad \implies \sqrt{(1-r_2)^2 -4r_1(1-r_1-r_2)\mathrm{sin}^2\left( \frac{\theta }{2}\right) } \le 1\\&\quad \implies (1-r_2)^2 \le 1 + 4r_1(1-r_1-r_2)\mathrm{sin}^2\left( \frac{\theta }{2}\right) \end{aligned}$$

Thus we obtain the Eq. (4.20). The inequality applies for all values of $\mathrm{sin}^2\left( \frac{\theta }{2}\right)$.

4.20 $$\begin{aligned} (1-r_2)^2 \le 1 + 4r_1(1-r_1-r_2)\mathrm{sin}^2\left( \frac{\theta }{2}\right) \end{aligned}$$

Substituting $\mathrm{sin}^2\left( \frac{\theta }{2}\right) = 0$ in Eq. (4.20) we get,

$$\begin{aligned}&(1-r_2)^2 \le 1 \\&\quad \implies -1 \le 1-r_2 \le 1 \\&\quad \implies 0 \le r_2 \le 2 \end{aligned}$$

Hence the range of $r_2$ is obtained as

4.21 $$\begin{aligned} 0 \le r_2 \le 2 \end{aligned}$$

Putting $\mathrm{sin}^2\left( \frac{\theta }{2}\right) = 1$ in Eq. (4.20) we get,

$$\begin{aligned}&(1-r_2)^2 \le 1 + 4r_1(1-r_1-r_2) \\&\quad \implies ((1-r_2-r_1)+r_1)^2 - 4r_1(1-r_1-r_2) \le 1 \\&\quad \implies (1-r_2-2r_1)^2 \le 1\\&\quad \implies -1 \le 1-r_2-2r_1 \le 1 \\&\quad \implies -r_2 \le 2r_1 \le 2 - r_2 \end{aligned}$$

Thus the Eq. (4.22) defining relation between $r_1$ and $r_2$ is obtained as

4.22 $$\begin{aligned} -r_2 \le 2r_1 \le 2 - r_2 \end{aligned}$$

But there is an additional condition that $r_1\ge 0$. By considering this condition, we get the triangular region with boundaries defined by Eqs. (4.23a)–(4.23c).

4.23a $$\begin{aligned}&r_1 = 0 \end{aligned}$$

4.23b $$\begin{aligned}&r_2 = 0 \end{aligned}$$

4.23c $$\begin{aligned}&r_2 + 2r_1 = 2 \end{aligned}$$

But as $X_{s,j}-X_{i,j}$ can be negative, $r_1$ can be replaced with $-r_1$. The region obtained by replacing with $-r_1$ has boundaries defined by

4.24a $$\begin{aligned}&r_1 = 0 \end{aligned}$$

4.24b $$\begin{aligned}&r_2 = 0 \end{aligned}$$

4.24c $$\begin{aligned}&r_2 + 2r_1= 2 \end{aligned}$$

**Fig. 7:**
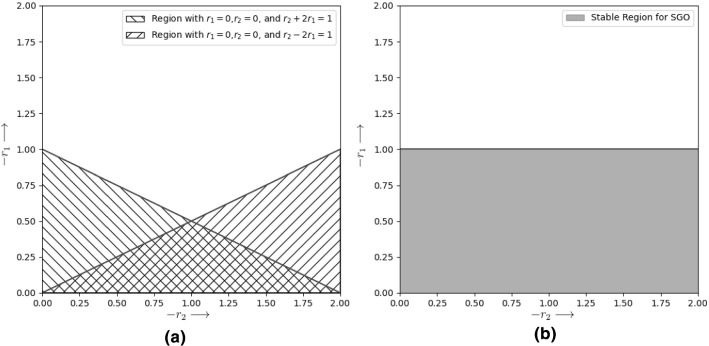
Graph demonstrating stable region for SGO w.r.t $r_1$ and $r_2$

So the complete region for $r_1$ and $r_2$, as shown in Fig. 7a, is equal to the union of the regions defined by Eqs. (4.23a)–(4.23c) and Eqs. (4.24a)–(4.24c). For ease of calculation, this region has been approximated by the rectangular region bounded by the constraint $0\le r_1\le 1$ and $0\le r_2\le 1$ as shown in Fig. 7b.

The calculations for Eq. (4.14) is similar and by following the same steps which was done for Eq. (4.15), we obtain the equation as

4.25 $$\begin{aligned} e^{-\hbox {J}\beta _m \Delta \tau } =&(1+r_1-r_2) - r_1*e^{\hbox {J}\theta }, \text { where } \nonumber \\&\quad \theta = \alpha _m(\pm a)\Delta \chi \end{aligned}$$

As per von neumann stability criterion $|e^{\hbox {J}\beta _m\Delta \tau }| \le 1$. So,

$$\begin{aligned}&\implies |(1+r_1-r_2) - r_1*e^{\hbox {J}\theta }| \le 1 \\&\quad \implies (1-r_2)^2 \le 1 - 4r_1(1+r_1-r_2)\mathrm{sin}^2\left( \frac{\theta }{2}\right) \end{aligned}$$

Thus we obtain the inequality

4.26 $$\begin{aligned} (1-r_2)^2 \le 1 + 4r_1(1-r_1-r_2)\mathrm{sin}^2\left( \frac{\theta }{2}\right) \end{aligned}$$

The inequality applies for all values of $\mathrm{sin}^2\left( \frac{\theta }{2}\right)$. Putting $\mathrm{sin}^2\left( \frac{\theta }{2}\right) = 0$ in Eq. (4.26) gives us the same condition as in Eq. (4.22). Putting $\mathrm{sin}^2\left( \frac{\theta }{2}\right) = 1$ in Eq. (4.20) we get,

$$\begin{aligned}&(1-r_2)^2 \le 1 - 4r_1(1+r_1-r_2) \\&\quad \implies ((1-r_2+r_1)-r_1)^2 + 4r_1(1+r_1-r_2) \le 1 \\&\quad \implies (1-r_2+2r_1)^2 \le 1\\&\quad \implies -1 \le 1-r_2+2r_1 \le 1 \\&\quad \implies r_2-2 \le 2r_1 \le r_2 \end{aligned}$$

Thus the resulting inequality is

4.27 $$\begin{aligned} -r_2 \le 2r_1 \le 2 - r_2 \end{aligned}$$

Using the condition that $r_1\ge 0$ with Eq. (4.27), the triangular region with boundaries defined by the lines Eqs. (4.24a)–(4.24c) is obtained. Again, as done before, replacing $r_1$ with $-r_1$, the region bounded by the lines is defined by Eqs. (4.23a)–(4.23c). So for Eq. (4.14) the same rectangular region as Eq. (4.14), which is bounded by the constraint $0\le r_1\le 1$ and $0\le r_2\le 1$ is obtained.

Thus for acquiring phase the stable range for $r_1$ and $r_2$ is obtained as $r_1 \in [0,1]$ and $r_2 \in [0,2]$.

**Experimental results**

**Fig. 8:**
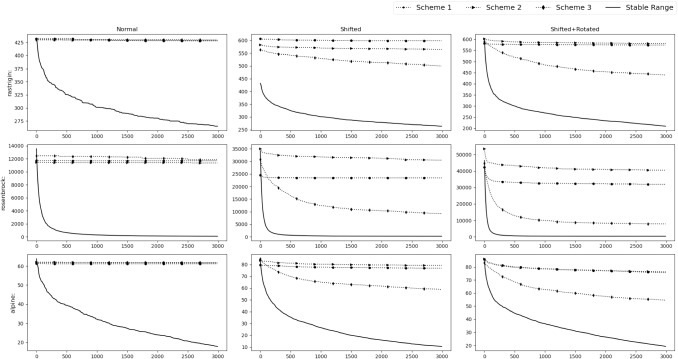
Experimental results of testing of the acquiring phase

To verify our findings, we remove the acquiring phase from SGO algorithm and only keep the improving phase. The stable range of *r* is compared against 3 schemes of unstable ranges of *r*, i.e.

(i) $r_1\in [1,2]$, $r_2\in [0,2]$ ($r_1$ outside stable range)
(ii) $r_1\in [0,1]$, $r_2\in [2,4]$ ($r_2$ outside stable range)
(iii) $r_1\in [1,1.5]$, $r_2\in [2,3]$ (Both outside stable range)

We have used Rastrigin, Rosenbrock and Alpine function for the testing with $D=30$, number of swarm members i.e. $N=50$ with 300 epochs (or $50*300=15{,}000$ function evaluations). The testing is done in 3 phases.

- *M*,*o* as identity matrix and null vector respectively. Average and Standard deviation taken over 30 iterations.
- *M* as identity matrix and *o* as a random vector whose value will be changed after each 10th iteration. Average and Standard deviation taken over 100 iterations.
- *M* as random rotation matrix and *o* as a random vector. Their values will be changed after each 12th iteration. Average and Standard deviation taken over 120 iterations.

Figure 8 shows the results of the 3 phases mentioned above. We can clearly see that the stable range performs better than the 3 unstable schemes.

**Table 2:** Result of optimization (10D default functions)

Name	Default range		Stable range		Unstable range	
	$r\in [0,1]$		$r\in [c-1,c+1]$		$r\in [c-2,c-1]\cup [c+1,c+2]$	
	$r_1,r_2\in [0,1]$		$r_1\in [0,1],r_2\in [0,2]$		$r_1\in [1,1.5],r_2\in [2,3]$	
Sphere	**C:0.2**	$\varvec{4.042e-138\pm 4.3e-138}$	**C:0.2**	$2.933e-136\pm 8.4e-137$	**C:0.2**	$3.172e-136\pm 6.1e-137$
	**C:0.4**	$1.735e-77\pm 2.4e-77$	**C:0.4**	$\varvec{5.762e-78\pm 8.6e-78}$	**C:0.4**	$4.907e-76\pm 1.2e-76$
	**C:0.6**	$1.833e-41\pm 9.9e-42$	**C:0.6**	$\varvec{8.766e-48\pm 2.6e-47}$	**C:0.6**	$8.222e-41\pm 1.4e-41$
	**C:0.8**	$1.439e-16\pm 9.5e-17$	**C:0.8**	$\varvec{7.427e-19\pm 1.8e-18}$	**C:0.8**	$7.698e-16\pm 2e-16$
	**C:1.0**	$2015\pm 1.2e+03$	**C:1.0**	$\varvec{259.9\pm 4.5e+02}$	**C:1.0**	$2.003e+04\pm 3.8e+03$
Rosenbrock	**C:0.2**	$\varvec{0\pm 0}$	**C:0.2**	$\varvec{0\pm 0}$	**C:0.2**	$\varvec{0\pm 0}$
	**C:0.4**	$\varvec{0\pm 0}$	**C:0.4**	$\varvec{0\pm 0}$	**C:0.4**	$\varvec{0\pm 0}$
	**C:0.6**	$1.115e-30\pm 2.6e-30$	**C:0.6**	$0.2218\pm 1.2$	**C:0.6**	$\varvec{6.59e-31\pm 2e-30}$
	**C:0.8**	$\varvec{3.777e-17\pm 2e-17}$	**C:0.8**	$0.1563\pm 0.84$	**C:0.8**	$1.754e-16\pm 7.7e-17$
	**C:1.0**	$209.1\pm 2e+02$	**C:1.0**	$\varvec{110\pm 78}$	**C:1.0**	$1533\pm 6.1e+02$
Ackley	**C:0.2**	$\varvec{0\pm 0}$	**C:0.2**	$\varvec{0\pm 0}$	**C:0.2**	$\varvec{0\pm 0}$
	**C:0.4**	$2.605e-15\pm 1.6e-15$	**C:0.4**	$\varvec{4.737e-16\pm 1.2e-15}$	**C:0.4**	$2.013e-15\pm 1.8e-15$
	**C:0.6**	$4.145e-15\pm 1.3e-15$	**C:0.6**	$\varvec{2.842e-15\pm 1.4e-15}$	**C:0.6**	$0.6656\pm 3.6$
	**C:0.8**	$\varvec{5.519e-09\pm 1.5e-09}$	**C:0.8**	$0.1891\pm 0.57$	**C:0.8**	$1.996\pm 6$
	**C:1.0**	$12.75\pm 2$	**C:1.0**	$\varvec{11.69\pm 4.5}$	**C:1.0**	$19.83\pm 0.36$
Griewank	**C:0.2**	$0.003128\pm 0.017$	**C:0.2**	$\varvec{0\pm 0}$	**C:0.2**	$\varvec{0\pm 0}$
	**C:0.4**	$0.02456\pm 0.11$	**C:0.4**	$\varvec{0.002927\pm 0.0072}$	**C:0.4**	$0.2788\pm 0.46$
	**C:0.6**	$0.3743\pm 0.41$	**C:0.6**	$\varvec{0.08866\pm 0.18}$	**C:0.6**	$0.5341\pm 0.5$
	**C:0.8**	$0.8694\pm 0.32$	**C:0.8**	$\varvec{0.09183\pm 0.1}$	**C:0.8**	$0.862\pm 0.4$
	**C:1.0**	$29.56\pm 18$	**C:1.0**	$\varvec{4.479\pm 4}$	**C:1.0**	$173.9\pm 49$
Rastrigin	**C:0.2**	$\varvec{0\pm 0}$	**C:0.2**	$\varvec{0\pm 0}$	**C:0.2**	$3.665\pm 20$
	**C:0.4**	$5.64\pm 7.9$	**C:0.4**	$\varvec{1.07\pm 4.9}$	**C:0.4**	$57.45\pm 50$
	**C:0.6**	$13.48\pm 6.3$	**C:0.6**	$\varvec{10.13\pm 11}$	**C:0.6**	$80.54\pm 38$
	**C:0.8**	$20.32\pm 8.3$	**C:0.8**	$\varvec{17.7\pm 14}$	**C:0.8**	$115.9\pm 19$
	**C:1.0**	$\varvec{39.23\pm 14}$	**C:1.0**	$41.89\pm 18$	**C:1.0**	$128.1\pm 17$
Alpine	**C:0.2**	$\varvec{4.05e-71\pm 5.2e-71}$	**C:0.2**	$5.531e-70\pm 1.1e-70$	**C:0.2**	$5.034e-70\pm 1e-70$
	**C:0.4**	$1.124e-40\pm 7.2e-41$	**C:0.4**	$\varvec{7.371e-41\pm 9.1e-41}$	**C:0.4**	$6.4e-40\pm 1.2e-40$
	**C:0.6**	$0.02448\pm 0.089$	**C:0.6**	$\varvec{3.491e-07\pm 1.9e-06}$	**C:0.6**	$5.313\pm 6.8$
	**C:0.8**	$0.4768\pm 0.53$	**C:0.8**	$\varvec{0.2318\pm 0.63}$	**C:0.8**	$18\pm 2.8$
	**C:1.0**	$3.075\pm 1.2$	**C:1.0**	$\varvec{1.396\pm 2}$	**C:1.0**	$18.05\pm 2.9$
Sum of powers	**C:0.2**	$\varvec{1.321e-195\pm 0}$	**C:0.2**	$3.462e-143\pm 8.1e-143$	**C:0.2**	$2.134e-141\pm 2.5e-141$
	**C:0.4**	$\varvec{5.869e-138\pm 3.2e-137}$	**C:0.4**	$1.63e-123\pm 5.4e-123$	**C:0.4**	$2.893e-81\pm 4.4e-81$
	**C:0.6**	$1.776e-50\pm 8.8e-50$	**C:0.6**	$\varvec{4.994e-105\pm 1.9e-104}$	**C:0.6**	$6.261e-46\pm 6.3e-46$
	**C:0.8**	$1.27e-23\pm 6e-23$	**C:0.8**	$\varvec{7.06e-54\pm 3.8e-53}$	**C:0.8**	$4.975e-21\pm 7.8e-21$
	**C:1.0**	$0.01267\pm 0.024$	**C:1.0**	$\varvec{0.0003156\pm 0.0009}$	**C:1.0**	$0.5998\pm 0.28$
Zakharov	**C:0.2**	$\varvec{6.194e-138\pm 6.3e-138}$	**C:0.2**	$1.385e-137\pm 2.5e-137$	**C:0.2**	$7.322e-138\pm 2.8e-138$
	**C:0.4**	$\varvec{2.742e-78\pm 1.6e-78}$	**C:0.4**	$1.218e-77\pm 6.8e-78$	**C:0.4**	$1.677e-77\pm 2.9e-77$
	**C:0.6**	$4.847e-43\pm 4e-43$	**C:0.6**	$\varvec{9.801e-45\pm 1.8e-44}$	**C:0.6**	$3.194e-42\pm 3.1e-42$
	**C:0.8**	$4.191e-18\pm 2.9e-18$	**C:0.8**	$\varvec{2.046e-19\pm 4.4e-19}$	**C:0.8**	$3.608e-17\pm 2.3e-17$
	**C:1.0**	$77.2\pm 39$	**C:1.0**	$\varvec{72.33\pm 78}$	**C:1.0**	$469.5\pm 1.8e+02$

**Table 3:** Result of optimization (30D default functions)

Name	Default range		Stable range		Unstable range	
	$r\in [0,1]$		$r\in [c-1,c+1]$		$r\in [c-2,c-1]\cup [c+1,c+2]$	
	$r_1,r_2\in [0,1]$		$r_1\in [0,1],r_2\in [0,2]$		$r_1\in [1,1.5],r_2\in [2,3]$	
Sphere	**C:0.2**	$\varvec{0\pm 0}$	**C:0.2**	$\varvec{0\pm 0}$	**C:0.2**	$\varvec{0\pm 0}$
	**C:0.4**	$\varvec{8.148e-237\pm 0}$	**C:0.4**	$1.1e-234\pm 0$	**C:0.4**	$1.14e-234\pm 0$
	**C:0.6**	$6.179e-130\pm 2.1e-130$	**C:0.6**	$\varvec{1.786e-141\pm 3.2e-141}$	**C:0.6**	$5.095e-129\pm 4.3e-130$
	**C:0.8**	$5.035e-55\pm 1.5e-55$	**C:0.8**	$\varvec{3.313e-58\pm 1.8e-57}$	**C:0.8**	$4.691e-54\pm 4.1e-55$
	**C:1.0**	$4405\pm 9.9e+02$	**C:1.0**	$\varvec{307.4\pm 2.7e+02}$	**C:1.0**	$6.491e+04\pm 6.6e+03$
Rosenbrock	**C:0.2**	$\varvec{0\pm 0}$	**C:0.2**	$\varvec{0\pm 0}$	**C:0.2**	$\varvec{0\pm 0}$
	**C:0.4**	$\varvec{0\pm 0}$	**C:0.4**	$\varvec{0\pm 0}$	**C:0.4**	$\varvec{0\pm 0}$
	**C:0.6**	$5.777e-31\pm 2.3e-30$	**C:0.6**	$\varvec{0\pm 0}$	**C:0.6**	$\varvec{0\pm 0}$
	**C:0.8**	$2.12e-29\pm 2.3e-29$	**C:0.8**	$2.336e-29\pm 2.2e-29$	**C:0.8**	$\varvec{1.681e-29\pm 2.8e-29}$
	**C:1.0**	$516.5\pm 2.1e+02$	**C:1.0**	$\varvec{362.8\pm 2.1e+02}$	**C:1.0**	$9842\pm 2.7e+03$
Ackley	**C:0.2**	$\varvec{0\pm 0}$	**C:0.2**	$\varvec{0\pm 0}$	**C:0.2**	$\varvec{0\pm 0}$
	**C:0.4**	$2.605e-15\pm 1.6e-15$	**C:0.4**	$\varvec{7.105e-16\pm 1.4e-15}$	**C:0.4**	$2.487e-15\pm 1.6e-15$
	**C:0.6**	$3.671e-15\pm 6.4e-16$	**C:0.6**	$\varvec{3.553e-15\pm 0}$	**C:0.6**	$3.79e-15\pm 8.9e-16$
	**C:0.8**	$9.237e-15\pm 3.3e-15$	**C:0.8**	$\varvec{8.053e-15\pm 2.9e-15}$	**C:0.8**	$5.323\pm 8.8$
	**C:1.0**	$12.29\pm 1$	**C:1.0**	$\varvec{12.05\pm 2.9}$	**C:1.0**	$19.96\pm 0.0014$
Griewank	**C:0.2**	$\varvec{0\pm 0}$	**C:0.2**	$\varvec{0\pm 0}$	**C:0.2**	$\varvec{0\pm 0}$
	**C:0.4**	$0.0003399\pm 0.0018$	**C:0.4**	$\varvec{0\pm 0}$	**C:0.4**	$0.03509\pm 0.19$
	**C:0.6**	$0.005177\pm 0.018$	**C:0.6**	$\varvec{0.001802\pm 0.0077}$	**C:0.6**	$0.138\pm 0.35$
	**C:0.8**	$0.02604\pm 0.076$	**C:0.8**	$\varvec{0.01272\pm 0.015}$	**C:0.8**	$0.2734\pm 0.45$
	**C:1.0**	$46.76\pm 13$	**C:1.0**	$\varvec{6.1\pm 6.7}$	**C:1.0**	$574.2\pm 59$
Rastrigin	**C:0.2**	$\varvec{0\pm 0}$	**C:0.2**	$\varvec{0\pm 0}$	**C:0.2**	$\varvec{0\pm 0}$
	**C:0.4**	$2.457\pm 7.6$	**C:0.4**	$\varvec{0\pm 0}$	**C:0.4**	$191.2\pm 1.5e+02$
	**C:0.6**	$17.29\pm 13$	**C:0.6**	$\varvec{6.687\pm 28}$	**C:0.6**	$296.5\pm 93$
	**C:0.8**	$\varvec{28.29\pm 10}$	**C:0.8**	$45.57\pm 38$	**C:0.8**	$371.1\pm 50$
	**C:1.0**	$\varvec{106.5\pm 19}$	**C:1.0**	$156.3\pm 31$	**C:1.0**	$432.5\pm 17$
Alpine	**C:0.2**	$2.883e-209\pm 0$	**C:0.2**	$2.68e-209\pm 0$	**C:0.2**	$\varvec{2.595e-209\pm 0}$
	**C:0.4**	$\varvec{4.944e-120\pm 2.8e-120}$	**C:0.4**	$5.572e-119\pm 1.7e-119$	**C:0.4**	$5.177e-119\pm 6.2e-120$
	**C:0.6**	$1.354e-66\pm 2.8e-67$	**C:0.6**	$\varvec{6.703e-73\pm 7e-73}$	**C:0.6**	$4.55\pm 13$
	**C:0.8**	$\varvec{0.02008\pm 0.06}$	**C:0.8**	$0.1081\pm 0.54$	**C:0.8**	$56.9\pm 15$
	**C:1.0**	$9.763\pm 2.5$	**C:1.0**	$\varvec{4.77\pm 4.4}$	**C:1.0**	$63.39\pm 5.2$
sum of powers	**C:0.2**	$\varvec{0\pm 0}$	**C:0.2**	$\varvec{0\pm 0}$	**C:0.2**	$\varvec{0\pm 0}$
	**C:0.4**	$\varvec{0\pm 0}$	**C:0.4**	$\varvec{0\pm 0}$	**C:0.4**	$1.628e-240\pm 0$
	**C:0.6**	$1.314e-185\pm 0$	**C:0.6**	$\varvec{0\pm 0}$	**C:0.6**	$9.934e-135\pm 9.9e-135$
	**C:0.8**	$8.053e-71\pm 1.9e-70$	**C:0.8**	$\varvec{2.358e-287\pm 0}$	**C:0.8**	$9.611e-60\pm 8.6e-60$
	**C:1.0**	$4.438e-05\pm 5.8e-05$	**C:1.0**	$\varvec{6.563e-08\pm 2.1e-07}$	**C:1.0**	$0.796\pm 0.22$
zakharov	**C:0.2**	$\varvec{0\pm 0}$	**C:0.2**	$\varvec{0\pm 0}$	**C:0.2**	$\varvec{0\pm 0}$
	**C:0.4**	$\varvec{7.831e-238\pm 0}$	**C:0.4**	$2.509e-236\pm 0$	**C:0.4**	$2.188e-236\pm 0$
	**C:0.6**	$\varvec{9.289e-132\pm 1.1e-131}$	**C:0.6**	$9.312e-131\pm 5.3e-131$	**C:0.6**	$2.081e-130\pm 2.6e-130$
	**C:0.8**	$\varvec{2.87e-58\pm 1e-57}$	**C:0.8**	$8.57e-58\pm 1.6e-57$	**C:0.8**	$1.236e-55\pm 1e-55$
	**C:1.0**	$\varvec{37.47\pm 19}$	**C:1.0**	$166.4\pm 1.3e+02$	**C:1.0**	$1506\pm 4.3e+02$

**Table 4:** Result of optimization (10D shifted functions)

Name	Default range		Stable range		Unstable range	
	$r\in [0,1]$		$r\in [c-1,c+1]$		$r\in [c-2,c-1]\cup [c+1,c+2]$	
	$r_1,r_2\in [0,1]$		$r_1\in [0,1],r_2\in [0,2]$		$r_1\in [1,1.5],r_2\in [2,3]$	
Sphere	**C:0.2**	$3027\pm 2.9e+03$	**C:0.2**	$\varvec{366.9\pm 6e+02}$	**C:0.2**	$1.629e+04\pm 5.1e+03$
	**C:0.4**	$4219\pm 3.5e+03$	**C:0.4**	$\varvec{90.55\pm 2.8e+02}$	**C:0.4**	$1.401e+04\pm 5.4e+03$
	**C:0.6**	$4674\pm 3.2e+03$	**C:0.6**	$\varvec{83.06\pm 2.6e+02}$	**C:0.6**	$1.339e+04\pm 5.6e+03$
	**C:0.8**	$5085\pm 3.6e+03$	**C:0.8**	$\varvec{4.261\pm 13}$	**C:0.8**	$1.327e+04\pm 4.9e+03$
	**C:1.0**	$8703\pm 5e+03$	**C:1.0**	$\varvec{1232\pm 1.6e+03}$	**C:1.0**	$1.953e+04\pm 5.6e+03$
Rosenbrock	**C:0.2**	$321\pm 4.6e+02$	**C:0.2**	$\varvec{69.21\pm 1.3e+02}$	**C:0.2**	$1565\pm 9.3e+02$
	**C:0.4**	$367.1\pm 4.5e+02$	**C:0.4**	$\varvec{39.98\pm 49}$	**C:0.4**	$1332\pm 7.4e+02$
	**C:0.6**	$482.6\pm 5.8e+02$	**C:0.6**	$\varvec{37.38\pm 45}$	**C:0.6**	$885.1\pm 4.9e+02$
	**C:0.8**	$618.2\pm 9.2e+02$	**C:0.8**	$\varvec{37.73\pm 40}$	**C:0.8**	$884.5\pm 5.6e+02$
	**C:1.0**	$1419\pm 1.5e+03$	**C:1.0**	$\varvec{187.9\pm 2.8e+02}$	**C:1.0**	$1695\pm 1e+03$
Ackley	**C:0.2**	$15.6\pm 3.3$	**C:0.2**	$\varvec{7.754\pm 5.5}$	**C:0.2**	$19.88\pm 0.79$
	**C:0.4**	$16.34\pm 3.2$	**C:0.4**	$\varvec{7.513\pm 5.5}$	**C:0.4**	$19.54\pm 0.8$
	**C:0.6**	$16.52\pm 3.3$	**C:0.6**	$\varvec{7.78\pm 6.4}$	**C:0.6**	$19.13\pm 1$
	**C:0.8**	$17.21\pm 2.9$	**C:0.8**	$\varvec{7.489\pm 6.8}$	**C:0.8**	$18.92\pm 1.6$
	**C:1.0**	$17.34\pm 2.4$	**C:1.0**	$\varvec{14.36\pm 4.5}$	**C:1.0**	$19.83\pm 1$
Griewank	**C:0.2**	$34.41\pm 29$	**C:0.2**	$\varvec{3.27\pm 3.8}$	**C:0.2**	$132\pm 40$
	**C:0.4**	$39.38\pm 32$	**C:0.4**	$\varvec{2.564\pm 4}$	**C:0.4**	$121.9\pm 39$
	**C:0.6**	$48.84\pm 33$	**C:0.6**	$\varvec{1.257\pm 1.5}$	**C:0.6**	$118.4\pm 49$
	**C:0.8**	$43.49\pm 35$	**C:0.8**	$\varvec{1.299\pm 2.6}$	**C:0.8**	$108.9\pm 57$
	**C:1.0**	$82.74\pm 44$	**C:1.0**	$\varvec{15.55\pm 22}$	**C:1.0**	$167.5\pm 43$
Rastrigin	**C:0.2**	$57.92\pm 23$	**C:0.2**	$\varvec{47.78\pm 18}$	**C:0.2**	$124.6\pm 21$
	**C:0.4**	$58.92\pm 19$	**C:0.4**	$\varvec{50.39\pm 18}$	**C:0.4**	$120.3\pm 19$
	**C:0.6**	$61.83\pm 25$	**C:0.6**	$\varvec{45.84\pm 17}$	**C:0.6**	$114.8\pm 20$
	**C:0.8**	$56.62\pm 24$	**C:0.8**	$\varvec{41.75\pm 15}$	**C:0.8**	$109\pm 24$
	**C:1.0**	$68.83\pm 24$	**C:1.0**	$\varvec{52.04\pm 21}$	**C:1.0**	$116.8\pm 21$
Alpine	**C:0.2**	$\varvec{2.039\pm 1.6}$	**C:0.2**	$2.692\pm 2.5$	**C:0.2**	$15.16\pm 2.8$
	**C:0.4**	$\varvec{1.762\pm 1.3}$	**C:0.4**	$2.825\pm 2.8$	**C:0.4**	$13.56\pm 3.2$
	**C:0.6**	$\varvec{2.279\pm 1.7}$	**C:0.6**	$2.378\pm 2.9$	**C:0.6**	$15.44\pm 3.5$
	**C:0.8**	$1.98\pm 1.5$	**C:0.8**	$\varvec{1.468\pm 2.3}$	**C:0.8**	$14.65\pm 4$
	**C:1.0**	$4.478\pm 1.9$	**C:1.0**	$\varvec{2.593\pm 2.1}$	**C:1.0**	$15.43\pm 3.3$
Sum of powers	**C:0.2**	$0.07342\pm 0.1$	**C:0.2**	$\varvec{0.0006466\pm 0.0019}$	**C:0.2**	$0.4429\pm 0.32$
	**C:0.4**	$0.09127\pm 0.16$	**C:0.4**	$\varvec{0.001545\pm 0.0082}$	**C:0.4**	$0.4041\pm 0.29$
	**C:0.6**	$0.1412\pm 0.18$	**C:0.6**	$\varvec{0.0006761\pm 0.0051}$	**C:0.6**	$0.438\pm 0.4$
	**C:0.8**	$0.1539\pm 0.23$	**C:0.8**	$\varvec{4.373e-06\pm 2.1e-05}$	**C:0.8**	$0.2851\pm 0.33$
	**C:1.0**	$0.2864\pm 0.41$	**C:1.0**	$\varvec{0.03304\pm 0.14}$	**C:1.0**	$0.8882\pm 1.2$
zakharov	**C:0.2**	$70.72\pm 75$	**C:0.2**	$\varvec{32.9\pm 44}$	**C:0.2**	$325.1\pm 1.2e+02$
	**C:0.4**	$96.77\pm 93$	**C:0.4**	$\varvec{22.92\pm 21}$	**C:0.4**	$260.9\pm 1.5e+02$
	**C:0.6**	$74.78\pm 62$	**C:0.6**	$\varvec{20.71\pm 29}$	**C:0.6**	$247.3\pm 1.2e+02$
	**C:0.8**	$112.2\pm 83$	**C:0.8**	$\varvec{6.52\pm 5.8}$	**C:0.8**	$227.4\pm 1.3e+02$
	**C:1.0**	$257.3\pm 1.5e+02$	**C:1.0**	$\varvec{132.8\pm 1.7e+02}$	**C:1.0**	$560.5\pm 3.1e+02$

**Table 5:** Result of optimization (30D shifted functions)

Name	Default range		Stable range		Unstable range	
	$r\in [0,1]$		$r\in [c-1,c+1]$		$r\in [c-2,c-1]\cup [c+1,c+2]$	
	$r_1,r_2\in [0,1]$		$r_1\in [0,1],r_2\in [0,2]$		$r_1\in [1,1.5],r_2\in [2,3]$	
Sphere	**C:0.2**	$\varvec{77.6\pm 2.1e+02}$	**C:0.2**	$390.2\pm 6.3e+02$	**C:0.2**	$8.633e+04\pm 1.4e+04$
	**C:0.4**	$\varvec{41.89\pm 1.1e+02}$	**C:0.4**	$207.9\pm 5.2e+02$	**C:0.4**	$8.061e+04\pm 1.4e+04$
	**C:0.6**	$\varvec{95.06\pm 2.5e+02}$	**C:0.6**	$123.3\pm 2.5e+02$	**C:0.6**	$7.681e+04\pm 1.5e+04$
	**C:0.8**	$135.5\pm 3.5e+02$	**C:0.8**	$\varvec{28.29\pm 69}$	**C:0.8**	$8.327e+04\pm 1.9e+04$
	**C:1.0**	$2.657e+04\pm 8.6e+03$	**C:1.0**	$\varvec{3391\pm 3.5e+03}$	**C:1.0**	$7.047e+04\pm 1.1e+04$
Rosenbrock	**C:0.2**	$154.9\pm 65$	**C:0.2**	$\varvec{131.5\pm 83}$	**C:0.2**	$1.83e+04\pm 5.7e+03$
	**C:0.4**	$148.3\pm 66$	**C:0.4**	$\varvec{132.4\pm 94}$	**C:0.4**	$1.781e+04\pm 7.2e+03$
	**C:0.6**	$149\pm 1.2e+02$	**C:0.6**	$\varvec{98.49\pm 50}$	**C:0.6**	$1.632e+04\pm 6.5e+03$
	**C:0.8**	$162.8\pm 73$	**C:0.8**	$\varvec{88.5\pm 45}$	**C:0.8**	$1.378e+04\pm 7.4e+03$
	**C:1.0**	$3719\pm 2e+03$	**C:1.0**	$\varvec{526.9\pm 4.7e+02}$	**C:1.0**	$1.07e+04\pm 4e+03$
Ackley	**C:0.2**	$14.99\pm 3.4$	**C:0.2**	$\varvec{5.736\pm 4}$	**C:0.2**	$20.88\pm 0.23$
	**C:0.4**	$16.05\pm 3$	**C:0.4**	$\varvec{5.402\pm 3.9}$	**C:0.4**	$20.85\pm 0.25$
	**C:0.6**	$16.1\pm 2.9$	**C:0.6**	$\varvec{4.728\pm 4.1}$	**C:0.6**	$20.71\pm 0.34$
	**C:0.8**	$17\pm 2.6$	**C:0.8**	$\varvec{3.782\pm 6.3}$	**C:0.8**	$20.64\pm 0.54$
	**C:1.0**	$18.42\pm 0.98$	**C:1.0**	$\varvec{15.7\pm 3.5}$	**C:1.0**	$20.93\pm 0.25$
Griewank	**C:0.2**	$\varvec{1.029\pm 0.65}$	**C:0.2**	$4.266\pm 6.7$	**C:0.2**	$757.1\pm 1.2e+02$
	**C:0.4**	$\varvec{2.702\pm 9}$	**C:0.4**	$3.707\pm 5.7$	**C:0.4**	$703.1\pm 1.3e+02$
	**C:0.6**	$\varvec{1.852\pm 2.5}$	**C:0.6**	$2.472\pm 3.2$	**C:0.6**	$714.7\pm 1.2e+02$
	**C:0.8**	$1.712\pm 1.7$	**C:0.8**	$\varvec{0.5808\pm 0.59}$	**C:0.8**	$712.2\pm 1.5e+02$
	**C:1.0**	$216.5\pm 65$	**C:1.0**	$\varvec{34.43\pm 44}$	**C:1.0**	$625.5\pm 1.2e+02$
Rastrigin	**C:0.2**	$\varvec{163.7\pm 37}$	**C:0.2**	$210.5\pm 48$	**C:0.2**	$489.9\pm 31$
	**C:0.4**	$\varvec{187.2\pm 43}$	**C:0.4**	$215.6\pm 47$	**C:0.4**	$481\pm 48$
	**C:0.6**	$\varvec{179\pm 35}$	**C:0.6**	$213.2\pm 49$	**C:0.6**	$469.1\pm 46$
	**C:0.8**	$\varvec{163.9\pm 37}$	**C:0.8**	$186.4\pm 46$	**C:0.8**	$455.1\pm 60$
	**C:1.0**	$199.2\pm 36$	**C:1.0**	$\varvec{168.9\pm 42}$	**C:1.0**	$451\pm 43$
Alpine	**C:0.2**	$\varvec{0.906\pm 1.1}$	**C:0.2**	$11.17\pm 8.6$	**C:0.2**	$66.76\pm 8.1$
	**C:0.4**	$\varvec{0.6791\pm 0.89}$	**C:0.4**	$12.48\pm 10$	**C:0.4**	$63.11\pm 8.5$
	**C:0.6**	$\varvec{0.9522\pm 1}$	**C:0.6**	$10.28\pm 8.4$	**C:0.6**	$61.82\pm 7.8$
	**C:0.8**	$\varvec{0.6743\pm 0.89}$	**C:0.8**	$6.801\pm 6.6$	**C:0.8**	$62.73\pm 8.2$
	**C:1.0**	$18.27\pm 4.1$	**C:1.0**	$\varvec{8.297\pm 4.6}$	**C:1.0**	$60.34\pm 10$
Sum of powers	**C:0.2**	$\varvec{1.08e-05\pm 8.5e-05}$	**C:0.2**	$0.0002463\pm 0.0014$	**C:0.2**	$1.878\pm 0.94$
	**C:0.4**	$\varvec{8.633e-07\pm 3.8e-06}$	**C:0.4**	$6.075e-06\pm 2.6e-05$	**C:0.4**	$2.552\pm 0.91$
	**C:0.6**	$\varvec{1.116e-05\pm 3.9e-05}$	**C:0.6**	$1.256e-05\pm 9.6e-05$	**C:0.6**	$2.401\pm 1$
	**C:0.8**	$3.137e-06\pm 1e-05$	**C:0.8**	$\varvec{1.498e-06\pm 1.3e-05}$	**C:0.8**	$2.081\pm 1$
	**C:1.0**	$0.2864\pm 0.43$	**C:1.0**	$\varvec{0.04058\pm 0.21}$	**C:1.0**	$6.963\pm 9.3$
Zakharov	**C:0.2**	$\varvec{17.89\pm 14}$	**C:0.2**	$235.9\pm 71$	**C:0.2**	$1308\pm 2.4e+02$
	**C:0.4**	$\varvec{15.9\pm 14}$	**C:0.4**	$191.2\pm 63$	**C:0.4**	$1281\pm 2.8e+02$
	**C:0.6**	$\varvec{24.19\pm 33}$	**C:0.6**	$170\pm 61$	**C:0.6**	$1148\pm 2.7e+02$
	**C:0.8**	$\varvec{17.14\pm 16}$	**C:0.8**	$99.43\pm 36$	**C:0.8**	$1134\pm 3.1e+02$
	**C:1.0**	$135.9\pm 76$	**C:1.0**	$\varvec{124.3\pm 1.4e+02}$	**C:1.0**	$1646\pm 3.7e+02$

**Table 6:** Result of optimization (10D shifted and rotated functions)

Name	Default range		Stable range		Unstable range	
	$r\in [0,1]$		$r\in [c-1,c+1]$		$r\in [c-2,c-1]\cup [c+1,c+2]$	
	$r_1,r_2\in [0,1]$		$r_1\in [0,1],r_2\in [0,2]$		$r_1\in [1,1.5],r_2\in [2,3]$	
Sphere	**C:0.2**	$4150\pm 3.7e+03$	**C:0.2**	$\varvec{152.7\pm 3.3e+02}$	**C:0.2**	$1.583e+04\pm 4.4e+03$
	**C:0.4**	$4741\pm 3.6e+03$	**C:0.4**	$\varvec{196.6\pm 4.3e+02}$	**C:0.4**	$1.316e+04\pm 4.3e+03$
	**C:0.6**	$4688\pm 4e+03$	**C:0.6**	$\varvec{73.86\pm 2e+02}$	**C:0.6**	$1.413e+04\pm 6.2e+03$
	**C:0.8**	$5886\pm 4.5e+03$	**C:0.8**	$\varvec{9.128\pm 31}$	**C:0.8**	$1.202e+04\pm 5.5e+03$
	**C:1.0**	$9112\pm 5.2e+03$	**C:1.0**	$\varvec{1170\pm 1.6e+03}$	**C:1.0**	$1.519e+04\pm 6.3e+03$
Rosenbrock	**C:0.2**	$441\pm 5.2e+02$	**C:0.2**	$\varvec{129.1\pm 2.2e+02}$	**C:0.2**	$2301\pm 1.5e+03$
	**C:0.4**	$680.6\pm 7.2e+02$	**C:0.4**	$\varvec{150.5\pm 2.1e+02}$	**C:0.4**	$1688\pm 8.5e+02$
	**C:0.6**	$742.5\pm 1e+03$	**C:0.6**	$\varvec{90.62\pm 1.2e+02}$	**C:0.6**	$1223\pm 7.4e+02$
	**C:0.8**	$1167\pm 1e+03$	**C:0.8**	$\varvec{105.7\pm 1.1e+02}$	**C:0.8**	$865.1\pm 6.6e+02$
	**C:1.0**	$2651\pm 2.7e+03$	**C:1.0**	$\varvec{269.4\pm 3.7e+02}$	**C:1.0**	$2126\pm 2.3e+03$
Ackley	**C:0.2**	$16.32\pm 3.4$	**C:0.2**	$\varvec{7.619\pm 5.1}$	**C:0.2**	$19.79\pm 0.64$
	**C:0.4**	$16.42\pm 3.4$	**C:0.4**	$\varvec{7.395\pm 5.5}$	**C:0.4**	$19.4\pm 1$
	**C:0.6**	$17.14\pm 2.8$	**C:0.6**	$\varvec{6.921\pm 6.1}$	**C:0.6**	$19.05\pm 1.3$
	**C:0.8**	$17.17\pm 3.4$	**C:0.8**	$\varvec{9.28\pm 6.8}$	**C:0.8**	$18.78\pm 1.4$
	**C:1.0**	$17.63\pm 2.3$	**C:1.0**	$\varvec{15.17\pm 4.7}$	**C:1.0**	$19.84\pm 0.73$
Griewank	**C:0.2**	$28\pm 22$	**C:0.2**	$\varvec{3.259\pm 5.9}$	**C:0.2**	$156.5\pm 40$
	**C:0.4**	$40.82\pm 36$	**C:0.4**	$\varvec{2.472\pm 3.2}$	**C:0.4**	$119.5\pm 40$
	**C:0.6**	$51.83\pm 40$	**C:0.6**	$\varvec{1.399\pm 1.4}$	**C:0.6**	$99.43\pm 49$
	**C:0.8**	$62.38\pm 48$	**C:0.8**	$\varvec{0.865\pm 0.56}$	**C:0.8**	$89.79\pm 51$
	**C:1.0**	$80.87\pm 49$	**C:1.0**	$\varvec{16.93\pm 29}$	**C:1.0**	$148.6\pm 43$
Rastrigin	**C:0.2**	$\varvec{60.08\pm 20}$	**C:0.2**	$60.45\pm 23$	**C:0.2**	$118.7\pm 19$
	**C:0.4**	$62.86\pm 23$	**C:0.4**	$\varvec{61.63\pm 19}$	**C:0.4**	$114.3\pm 20$
	**C:0.6**	$64.35\pm 22$	**C:0.6**	$\varvec{56.46\pm 18}$	**C:0.6**	$112.2\pm 19$
	**C:0.8**	$58.07\pm 20$	**C:0.8**	$\varvec{49.31\pm 19}$	**C:0.8**	$103\pm 21$
	**C:1.0**	$68.27\pm 24$	**C:1.0**	$\varvec{56.37\pm 25}$	**C:1.0**	$113\pm 19$
Alpine	**C:0.2**	$\varvec{3.978\pm 1.8}$	**C:0.2**	$4.701\pm 3$	**C:0.2**	$14.16\pm 2.9$
	**C:0.4**	$\varvec{3.314\pm 1.9}$	**C:0.4**	$5.029\pm 3.2$	**C:0.4**	$12.99\pm 2.3$
	**C:0.6**	$\varvec{3.742\pm 1.9}$	**C:0.6**	$4.314\pm 3.3$	**C:0.6**	$12.58\pm 3.1$
	**C:0.8**	$4.057\pm 2.5$	**C:0.8**	$\varvec{3.464\pm 3.1}$	**C:0.8**	$11.63\pm 2.9$
	**C:1.0**	$5.353\pm 2.3$	**C:1.0**	$\varvec{4.025\pm 2.4}$	**C:1.0**	$13.01\pm 3.5$
Sum of powers	**C:0.2**	$0.0389\pm 0.071$	**C:0.2**	$\varvec{0.001253\pm 0.0051}$	**C:0.2**	$0.433\pm 0.26$
	**C:0.4**	$0.05334\pm 0.21$	**C:0.4**	$\varvec{0.000174\pm 0.00089}$	**C:0.4**	$0.3124\pm 0.24$
	**C:0.6**	$0.05502\pm 0.11$	**C:0.6**	$\varvec{6.444e-05\pm 0.00046}$	**C:0.6**	$0.2639\pm 0.26$
	**C:0.8**	$0.1219\pm 0.27$	**C:0.8**	$\varvec{1.661e-05\pm 7.9e-05}$	**C:0.8**	$0.2353\pm 0.3$
	**C:1.0**	$0.1476\pm 0.25$	**C:1.0**	$\varvec{0.02807\pm 0.18}$	**C:1.0**	$0.6131\pm 0.63$
Zakharov	**C:0.2**	$112.8\pm 96$	**C:0.2**	$\varvec{35.11\pm 61}$	**C:0.2**	$361.1\pm 3.3e+02$
	**C:0.4**	$107\pm 1e+02$	**C:0.4**	$\varvec{30.75\pm 62}$	**C:0.4**	$299.9\pm 1.7e+02$
	**C:0.6**	$114.8\pm 1e+02$	**C:0.6**	$\varvec{18.94\pm 38}$	**C:0.6**	$252.1\pm 1.2e+02$
	**C:0.8**	$100.5\pm 78$	**C:0.8**	$\varvec{5.325\pm 7.4}$	**C:0.8**	$5767\pm 5.5e+04$
	**C:1.0**	$253.1\pm 1.4e+02$	**C:1.0**	$\varvec{112.1\pm 1.5e+02}$	**C:1.0**	$545\pm 3e+02$

**Table 7:** Result of optimization (30D shifted and rotated functions)

Name	Normal range		Stable range		Unstable range	
	$r\in [0,1]$		$r\in [c-1,c+1]$		$r\in [c-2,c-1]\cup [c+1,c+2]$	
	$r_1,r_2\in [0,1]$		$r_1\in [0,1],r_2\in [0,2]$		$r_1\in [1,1.5],r_2\in [2,3]$	
Sphere	**C:0.2**	$\varvec{44.55\pm 1.5e+02}$	**C:0.2**	$499.7\pm 8.7e+02$	**C:0.2**	$8.174e+04\pm 1.5e+04$
	**C:0.4**	$\varvec{50.19\pm 1.4e+02}$	**C:0.4**	$318.4\pm 5.5e+02$	**C:0.4**	$8.125e+04\pm 1.1e+04$
	**C:0.6**	$\varvec{60.07\pm 1.5e+02}$	**C:0.6**	$80.06\pm 1.8e+02$	**C:0.6**	$7.913e+04\pm 1.5e+04$
	**C:0.8**	$76.53\pm 1.8e+02$	**C:0.8**	$\varvec{28.51\pm 68}$	**C:0.8**	$8.128e+04\pm 1.9e+04$
	**C:1.0**	$2.672e+04\pm 8.5e+03$	**C:1.0**	$\varvec{3372\pm 3.3e+03}$	**C:1.0**	$7.654e+04\pm 1.4e+04$
Rosenbrock	**C:0.2**	$300.2\pm 1.6e+02$	**C:0.2**	$\varvec{218.1\pm 97}$	**C:0.2**	$2.025e+04\pm 7.1e+03$
	**C:0.4**	$\varvec{301.2\pm 1.3e+02}$	**C:0.4**	$319.3\pm 1.7e+02$	**C:0.4**	$1.825e+04\pm 6.3e+03$
	**C:0.6**	$\varvec{232.4\pm 75}$	**C:0.6**	$247.1\pm 1.2e+02$	**C:0.6**	$1.543e+04\pm 6.2e+03$
	**C:0.8**	$\varvec{255.5\pm 1.1e+02}$	**C:0.8**	$276.6\pm 1.5e+02$	**C:0.8**	$1.157e+04\pm 6.2e+03$
	**C:1.0**	$5432\pm 3.2e+03$	**C:1.0**	$\varvec{703.6\pm 4.9e+02}$	**C:1.0**	$1.255e+04\pm 4.9e+03$
Ackley	**C:0.2**	$13.54\pm 3.6$	**C:0.2**	$\varvec{5.819\pm 3.7}$	**C:0.2**	$20.83\pm 0.17$
	**C:0.4**	$14.91\pm 3.6$	**C:0.4**	$\varvec{5.07\pm 3.5}$	**C:0.4**	$20.76\pm 0.22$
	**C:0.6**	$15.53\pm 3.4$	**C:0.6**	$\varvec{3.752\pm 3.2}$	**C:0.6**	$20.69\pm 0.34$
	**C:0.8**	$15.27\pm 3.2$	**C:0.8**	$\varvec{3.137\pm 4.9}$	**C:0.8**	$20.47\pm 0.64$
	**C:1.0**	$18.34\pm 1$	**C:1.0**	$\varvec{15.87\pm 3.5}$	**C:1.0**	$20.83\pm 0.21$
Griewank	**C:0.2**	$\varvec{1.252\pm 0.87}$	**C:0.2**	$3.504\pm 4.8$	**C:0.2**	$781.7\pm 1.3e+02$
	**C:0.4**	$\varvec{1.443\pm 2}$	**C:0.4**	$4.617\pm 7.5$	**C:0.4**	$737.8\pm 1.4e+02$
	**C:0.6**	$1.905\pm 5$	**C:0.6**	$\varvec{1.698\pm 2.3}$	**C:0.6**	$706\pm 1.3e+02$
	**C:0.8**	$1.413\pm 1.3$	**C:0.8**	$\varvec{0.7584\pm 0.68}$	**C:0.8**	$719.5\pm 1.4e+02$
	**C:1.0**	$222.3\pm 75$	**C:1.0**	$\varvec{27.92\pm 28}$	**C:1.0**	$665.1\pm 1.1e+02$
Rastrigin	**C:0.2**	$\varvec{164.1\pm 36}$	**C:0.2**	$251.8\pm 54$	**C:0.2**	$514.2\pm 42$
	**C:0.4**	$\varvec{187.6\pm 40}$	**C:0.4**	$266.4\pm 47$	**C:0.4**	$483.4\pm 47$
	**C:0.6**	$\varvec{202.5\pm 35}$	**C:0.6**	$266.6\pm 40$	**C:0.6**	$469.1\pm 53$
	**C:0.8**	$\varvec{157.4\pm 32}$	**C:0.8**	$208.1\pm 55$	**C:0.8**	$448.3\pm 53$
	**C:1.0**	$214.3\pm 43$	**C:1.0**	$\varvec{182.3\pm 50}$	**C:1.0**	$431.8\pm 42$
Alpine	**C:0.2**	$\varvec{8.209\pm 3.3}$	**C:0.2**	$26.75\pm 13$	**C:0.2**	$66.29\pm 7.5$
	**C:0.4**	$\varvec{8.573\pm 3.2}$	**C:0.4**	$24.11\pm 11$	**C:0.4**	$63.25\pm 6.4$
	**C:0.6**	$\varvec{8.227\pm 3.3}$	**C:0.6**	$23.71\pm 12$	**C:0.6**	$60.11\pm 7.6$
	**C:0.8**	$\varvec{8.554\pm 3.6}$	**C:0.8**	$14.64\pm 11$	**C:0.8**	$56.65\pm 7.1$
	**C:1.0**	$21.24\pm 4.8$	**C:1.0**	$\varvec{11.81\pm 4.8}$	**C:1.0**	$56.12\pm 8.4$
Sum of powers	**C:0.2**	$\varvec{1.089e-06\pm 2.6e-06}$	**C:0.2**	$8.12e-06\pm 4.1e-05$	**C:0.2**	$7.027\pm 9.1$
	**C:0.4**	$\varvec{2.836e-06\pm 1.3e-05}$	**C:0.4**	$8.852e-06\pm 4.3e-05$	**C:0.4**	$4.929\pm 5.1$
	**C:0.6**	$8.458e-06\pm 7.4e-05$	**C:0.6**	$\varvec{2.259e-06\pm 4.7e-06}$	**C:0.6**	$5.645\pm 6.7$
	**C:0.8**	$1.965e-06\pm 3.2e-06$	**C:0.8**	$\varvec{8.893e-07\pm 1.1e-06}$	**C:0.8**	$4.328\pm 5.5$
	**C:1.0**	$0.1477\pm 0.26$	**C:1.0**	$\varvec{0.03755\pm 0.27}$	**C:1.0**	$7.039\pm 15$
Zakharov	**C:0.2**	$\varvec{15.93\pm 15}$	**C:0.2**	$230.9\pm 72$	**C:0.2**	$1358\pm 2.6e+02$
	**C:0.4**	$\varvec{19.02\pm 21}$	**C:0.4**	$233.8\pm 1.4e+02$	**C:0.4**	$1231\pm 2.7e+02$
	**C:0.6**	$\varvec{17.32\pm 28}$	**C:0.6**	$155.7\pm 56$	**C:0.6**	$1166\pm 2.7e+02$
	**C:0.8**	$\varvec{12.97\pm 15}$	**C:0.8**	$107.7\pm 52$	**C:0.8**	$1072\pm 2.8e+02$
	**C:1.0**	$147.7\pm 98$	**C:1.0**	$\varvec{146.2\pm 1.5e+02}$	**C:1.0**	$1619\pm 4.6e+02$

## Numerical experiments

All the simulations were carried out in Matlab R2016a on the system having Intel Core i7 2.67 GHz processor and 8 GB RAM.

**Fig. 9:**
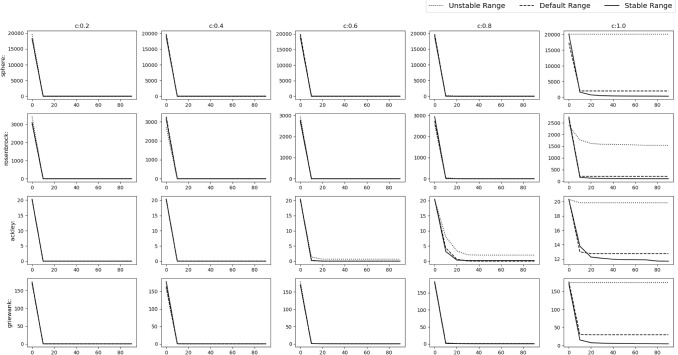
Experimental results of optimizing 10D Default functions. The solid,dashed and dotted lines denote stable range, default range and unstable range respectively

**Fig. 10:**
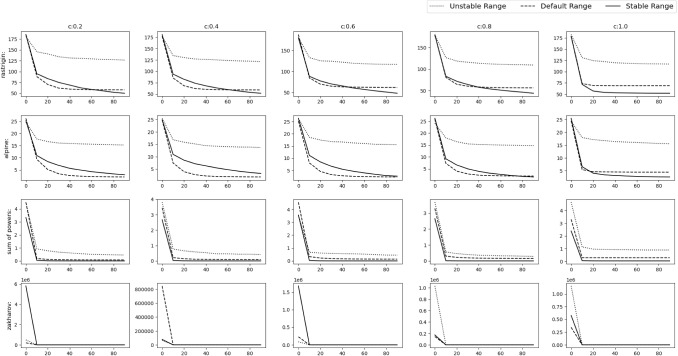
Experimental results of optimizing 10D Default functions. The solid, dashed and dotted lines denote stable range, default range and unstable range respectively

**Fig. 11:**
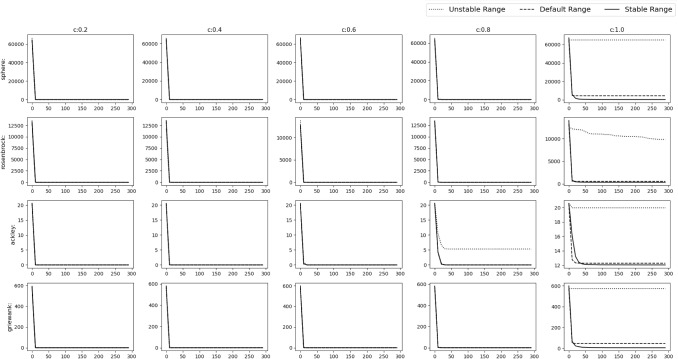
Experimental results of optimizing 30D Default functions. The solid, dashed and dotted lines denote stable range, default range and unstable range respectively

**Fig. 12:**
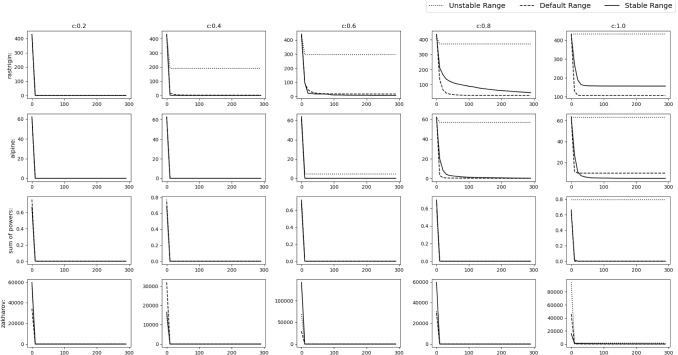
Experimental results of optimizing 30D Default functions. The solid, dashed and dotted lines denote stable range, default range and unstable range respectively

**Fig. 13:**
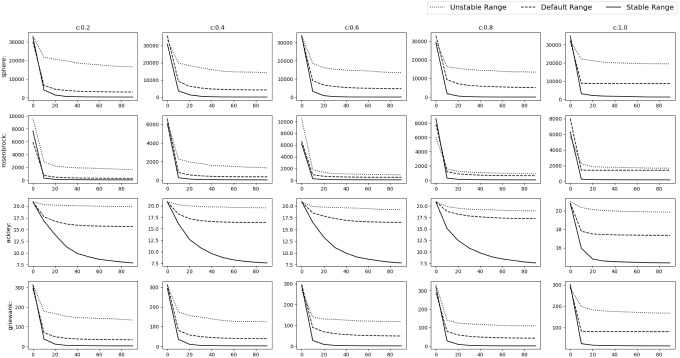
Experimental results of optimizing 10D Shifted functions. The solid, dashed and dotted lines denote stable range, default range and unstable range respectively

**Fig. 14:**
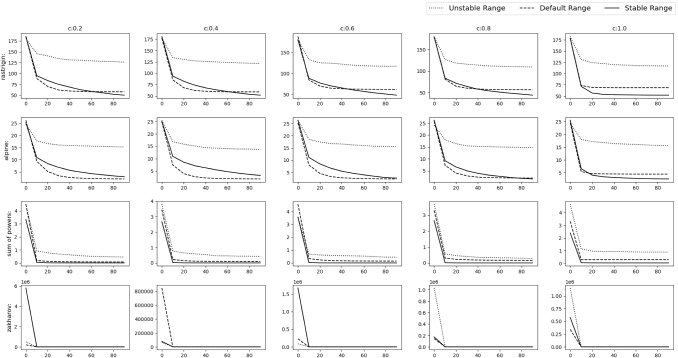
Experimental results of optimizing 10D Shifted functions. The solid, dashed and dotted lines denote stable range, default range and unstable range respectively

**Fig. 15:**
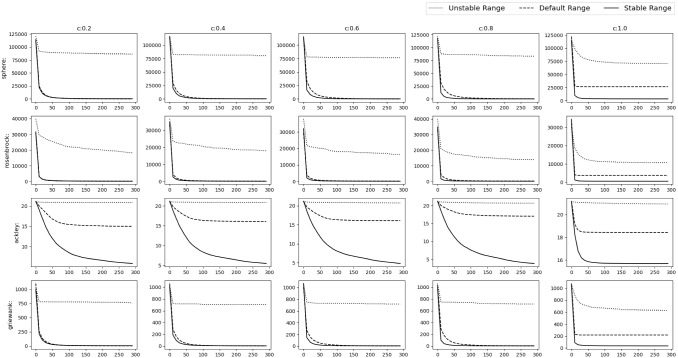
Experimental results of optimizing 30D Shifted functions. The solid,dashed and dotted lines denote stable range, default range and unstable range respectively

**Fig. 16:**
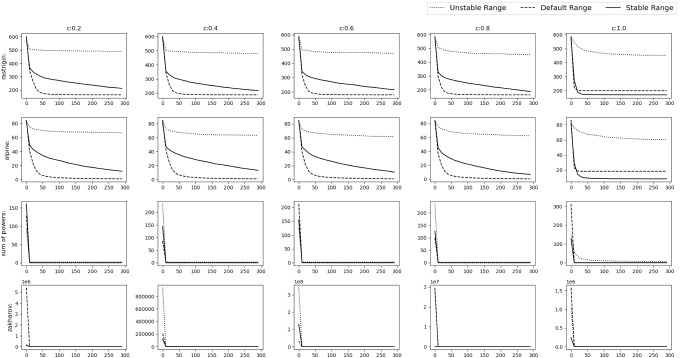
Experimental results of optimizing 30D Shifted functions. The solid, dashed and dotted lines denote stable range, default range and unstable range respectively

**Fig. 17:**
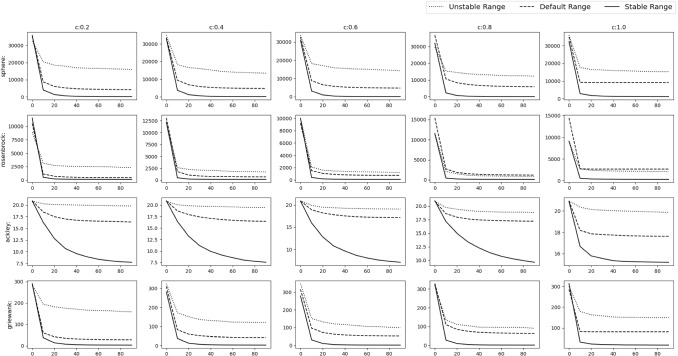
Experimental results of optimizing 10D Shifted and Rotated functions. The solid, dashed and dotted lines denote stable range, default range and unstable range respectively

**Fig. 18:**
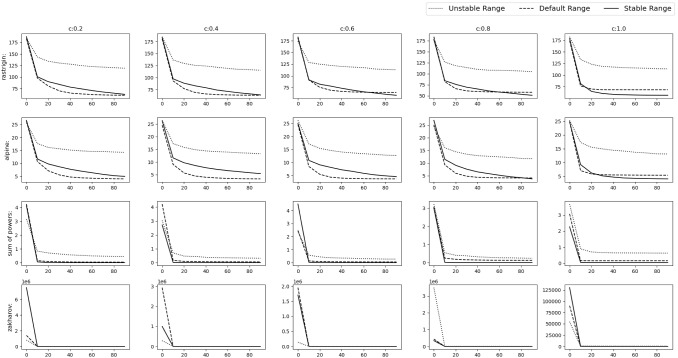
Experimental results of optimizing 10D Shifted and Rotated functions. The solid, dashed and dotted lines denote stable range, default range and unstable range respectively

**Fig. 19:**
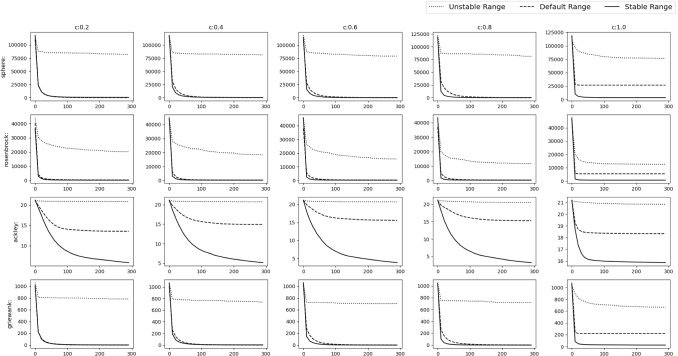
Experimental results of optimizing 30D Shifted and Rotated functions. The solid, dashed and dotted lines denote stable range, default range and unstable range respectively

**Fig. 20:**
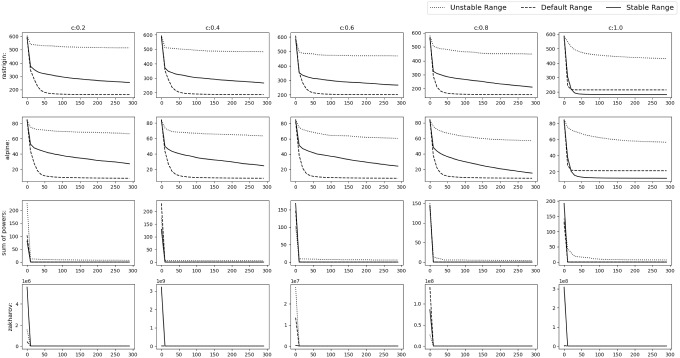
Experimental results of optimizing 30D Shifted and Rotated functions. The solid, dashed and dotted lines denote stable range, default range and unstable range respectively

To verify the findings, the performance of SGO algorithm (with both acquiring and improving phase) is tested with the ranges of $r,r_1,r_2$ found in the “Improving phase” section and “Acquiring phase” section. These ranges are referred to as stable range. We use $r\in [-c-2,-c-1] \, \cup \, [c+1,c+2]$ for the improving phase (defined in “Improving phase” section (b)) and $r_1\in [1,1.5]$, $r_2\in [2,3]$ for the acquiring phase (defined in “Acquiring phase” section (a)) to test the performance of

SGO when the parameters are outside of the stable range. These ranges are referred as Unstable Range. Finally the ranges of SGO mentioned in the original paper [3] is taken to test how well the stable range performs compared to the default ranges. These ranges are referred to as Default Range. The test is performed for 10D and 30D versions of the functions mentioned in Table 1 using $N=10$,epoch$=100$ and $N=50$, epoch$=300$ respectively. Values of *M* and *o* (“Optimization functions” section) are changed to test the optimization functions in different scenarios. In Tables 2, 3, 4, 5, 6, 7, the bold value represents the optimal value obtained out of three types of ranges mentioned above.

### Default numerical benchmark functions

This is the simplest scenario where the gbest or the *o* vector lies at the origin and no rotation is done i.e. *M* is the identity matrix. The results can be seen in Figs. 9, 10 and Table 2 for the 10D experiment and Figs. 11, 12 and Table 3 for the 30D experiment.

The results show that the default range and stable range perform nearly similar while the unstable range gives the worst results. These results are more apparent as the *c* value increases.

### Shifted Gbest numerical benchmark functions

In this scenario the gbest or the *o* vector is shifted to a different position randomly chosen inside the D-dimensional space defined by the range of $A_i$. No rotation is done i.e. *M* is the identity matrix. The results can be observed in Figs. 13, 14 and Table 4 for the 10D experiment and Figs. 15, 16 and Table 5 for the 30D experiment. The unstable range clearly gives the worst result. Between default range and stable range,the stable range converges a bit slower than default range in some cases, but it always goes on to find a better value than the default range in all the functions. The controlled randomness may be the reason. In most of the algorithms, it is assumed that the range of random parameters should be (0,1), but the relationship and dependencies among the parameter is ignored, which should not be the case.

### Shifted and rotated numerical benchmark functions

In this scenario the *gbest* or the *o* vector is shifted to a different position randomly chosen inside the D-dimensional space defined by the range of $A_i$. Random rotation is done i.e. *M* is set as a random D-dimensional rotation matrix. From Figs. 17, 18 and Table 6 could be observed that stable range performed better than the other unstable ranges for the 10D experiment and in Figs. 19, 20 and Table 7 for the 30D experiment same observations were obtained. Here also unstable range performed worst and stable range always converged.

## Conclusion

Stability analysis helps in determining the reliability factor of an algorithm. In this paper, Von Neumann stability analysis procedure was used to determine the appropriate range of parameters for which SGO algorithm always converges. The results were supported experimentally with suitable figures and tables. From the analysis, it is deduced that unstable range of parameters of an algorithm may degrade its performance and should be avoided. Moreover, dependencies and relationship between the parameters of an algorithm should be determined because they contribute a lot to the performance and stability analysis provides a way to solve the purpose. This theoretical analysis procedure could be combined with other experimental methodologies in determining the robustness of any algorithm and thus minimizing the chance of failure. Stability analysis of many recent algorithms such as monarch butterfly optimization (MBO) [23], earthworm optimization algorithm (EWA) [24], elephant herding optimization (EHO) [25], moth search (MS) algorithm [26], Slime mould algorithm (SMA) [27], Harris hawks optimization (HHO) [28] and Past Present Future optimization(PPF) [29] which claims to provide promising results experimentally, yet cannot be found in the literature. So, this paves a way for the researchers to work upon more such algorithms.
